# Low-Field, Benchtop NMR Spectroscopy as a Potential Tool for Point-of-Care Diagnostics of Metabolic Conditions: Validation, Protocols and Computational Models

**DOI:** 10.3390/ht8010002

**Published:** 2018-12-27

**Authors:** Benita C. Percival, Martin Grootveld, Miles Gibson, Yasan Osman, Marco Molinari, Fereshteh Jafari, Tarsem Sahota, Mark Martin, Federico Casanova, Melissa L. Mather, Mark Edgar, Jinit Masania, Philippe B. Wilson

**Affiliations:** 1 Leicester School of Pharmacy, De Montfort University, The Gateway, Leicester LE1 9BH, UK; benita.c.percival@dmu.ac.uk (B.C.P.); mgrootveld@dmu.ac.uk (M.G.); P11264662@my365.dmu.ac.uk (M.G.); P17195212@my365.dmu.ac.uk (Y.O.); Jafari.rcgld@gmail.com (F.J.); Tarsem.sahota@dmu.ac.uk (T.S.); jinit.masania@dmu.ac.uk (J.M.); 2 Department of Chemical Sciences, University of Huddersfield, Queensgate, Huddersfield HD1 3DH, UK; M.Molinari@hud.ac.uk; 3 Greater Manchester NHS Trust, Stepping Hill Hospital, Poplar Grove, Hazel Grove, Stockport SK2 7JE, UK; mark@setpoints.co.uk; 4 Magritek GmbH, Philipsstraße 8, 52068 Aachen, Germany; federico@magritek.com; 5 Department of Electrical and Electronic Engineering, University of Nottingham, University Park, Nottingham NG7 2RD, UK; Melissa.Mather@nottingham.ac.uk; 6 Department of Chemistry, University of Loughborough, Epinal Way, Loughborough LE11 3TU, UK; M.Edgar@lboro.ac.uk

**Keywords:** metabolomics, benchtop 60 MHz NMR analysis, biomarkers, biomolecules, validation, protocol, diabetes

## Abstract

Novel sensing technologies for liquid biopsies offer promising prospects for the early detection of metabolic conditions through omics techniques. Indeed, high-field nuclear magnetic resonance (NMR) facilities are routinely used for metabolomics investigations on a range of biofluids in order to rapidly recognise unusual metabolic patterns in patients suffering from a range of diseases. However, these techniques are restricted by the prohibitively large size and cost of such facilities, suggesting a possible role for smaller, low-field NMR instruments in biofluid analysis. Herein we describe selected biomolecule validation on a low-field benchtop NMR spectrometer (60 MHz), and present an associated protocol for the analysis of biofluids on compact NMR instruments. We successfully detect common markers of diabetic control at low-to-medium concentrations through optimised experiments, including α-glucose (≤2.8 mmol/L) and acetone (25 µmol/L), and additionally in readily accessible biofluids, particularly human urine. We present a combined protocol for the analysis of these biofluids with low-field NMR spectrometers for metabolomics applications, and offer a perspective on the future of this technique appealing to ‘point-of-care’ applications.

## 1. Introduction

Although now recognised as a powerful tool in translational medicine, the principles of metabolomics were arguably first described by ancient Chinese scholars, who used ants to evaluate the glucose level in the urine of diabetic patients [1]. The ancient Egyptian and Greek societies (circa 300 BC) developed this further to detect differences in the taste of urine as a means of disease diagnosis [2]. Etymologically derived from the Greek language words for *change*, and *body* or *rule*, metabolomics and metabonomics, respectively, involve the measurement of metabolic responses to perturbation; metabolomics is centred on measurements of the entire metabolome, whereas metabonomics concentrates on longitudinal changes across the metabolome ascribable to interventional stimuli [3].

The early metabolomics revolution effectively began with *De Statica Medicina*, published in 1614 by Santorio Santorio, who described his quantitative approach to modern medicine and the first systematic study of basal metabolism [4]. Both mass-spectrometric (MS) and nuclear magnetic resonance (NMR) strategies, two of the most common modern analytical techniques for metabolic studies, were first described in the early part of the 20th century; MS by J. J. Thomson and F Aston in 1913 [5], and NMR by Bloch and Purcell in 1946 [6]. Although not described as such, the first analytical measurement of metabolites was first published by Pauling in 1971 [7], before the pioneering work of Nicholson and Sadler in the 1980s [8,9], and Willmitzer in 1987 [10].

Metabolic profiling now includes the measurement of a range of biofluids, including blood plasma and serum, saliva, urine, knee-joint synovial fluid, semen and cerebrospinal fluid [11]. Indeed, biological media will vary in suitability for each disease/condition investigated, with a wide range and high volume of metabolic data being extracted from each approach [12]. For example, common high-field (HF) NMR metabolomics studies performed at operating frequencies of 600 MHz and above can detect more than 150 metabolites in human urine, and quantify almost 70 metabolites in human blood plasma or serum [3]. Moreover, with these practices becoming commonplace, tools and repositories such as the Human Metabolome database (HMDB) [13] and Metaboanalyst [14] have been developed to serve the ‘omics’ community.

With the rapidly expanding functional improvements in both NMR and MS techniques, additional avenues for the incorporation of metabolite profiling in medicine have arisen [15], based on the miniaturisation of these technologies [16], biomarker discovery [17], and prognostic monitoring [18]. Although MS and NMR are both considered standard techniques for metabolomics and metabonomics studies [19], this investigation will focus on the latter, and more specifically, low-field (LF) compact, or mobile, benchtop NMR facilities. In general, these instruments operate at frequencies below 100 MHz, and are based on permanent magnets as opposed to the large, HF superconducting ones commonly found in analytical characterisation suites [20]. These facilities operating at LF suffer from the same issues of sensitivity which plagued early designs of NMR spectrometers [21]; however, with the employment of approaches such as solvent suppression [22] and magnet arrays [23], it is possible to simultaneously observe and monitor 20 or more metabolites in human saliva and urine at LF, as demonstrated here for the first time. Although this limits the detection capabilities of NMR when compared to those of larger, high-field instruments, the advantages of more compact, mobile NMR instruments, incorporating applications of chemometric/metabolomics approaches to the multivariate analysis of complex mixtures, have been demonstrated in fields such as materials science [24], forensic chemistry [25], chemical education [26] and biomedical sciences in general.

Herein, we present an updated protocol for the analysis of biofluids through compact, benchtop NMR measurements. Comprehensive protocols for metabolite measurement and profiling are currently available, and these rely on HF NMR or LC/MS techniques, such as that described in the pioneering works of Nicholson et al. [27]. We describe a complete procedural development for the analysis of biofluids by LF NMR analysis, including validation and quantification experiments, experimental guidelines, and metabolomics data analysis, and these are experimentally demonstrated by an appropriate example-based in the area of type 2 diabetes.

## 2. Materials and Reagents

All materials were purchased from Sigma-Aldrich Ltd. (Gillingham, UK), unless otherwise stated. 5-mm diameter NMR tubes were purchased from Norell (Morganton, NC, USA). Sodium phosphate monobasic (99%) for analysis (anhydrous) and sodium phosphate, dibasic, heptahydrate 99%+ for analysis were purchased from Acros Organics, Fisher Scientific, (Loughborough, UK). Eppendorf micropipettes and tips were purchased from Eppendorf (Stevenage, UK), and sterile universal containers were purchased from Starlab Ltd. (Milton Keynes, UK).

## 3. Equipment

A 60 MHz Magritek Spinsolve Benchtop NMR spectrometer was utilised for these work-tasks (similar instruments may be employed: Pulsar from Oxford Instruments (Abingdon, UK), NMReady from Nanalysis (Calgary, Canada), and PicoSpin from ThermoFisher Scientific (Waltham, MA, USA)). A Bruker (Billerica, MA, USA) 400 MHz Avance-1 NMR spectrometer fitted with a Quadruple nucleus probe was also employed for comparative investigations. We also acquired ^1^H NMR spectra on human urine samples on a JEOL (Tokyo, Japan) ECS-400 spectrometer (similar spectrometers can be utilised: e.g., JEOL or Bruker 400–700 MHz operating frequencies), as can Centrifuges/Rotamixers.

### ^1^H NMR Spectral Acquisition

Human urine samples in 5-mm diameter NMR tubes were inserted into the LF 60 MHz NMR instrument manually with an approximate acquisition period of 10 min. However, with recent advancements, automation is now a possibility for benchtop instrumentation through the use of a robotic arm autosampler. This allows for a larger number of samples to be prepared, and a reduction in periods of commonplace, onerous ‘hands-on’ user activity between sampling.

^1^H NMR spectra were acquired on a 60 MHz Magritek Spinsolve Ultra Benchtop system (Leicester School of Pharmacy, De Montfort University, Leicester, UK). This instrument provides the option to shim to sample or shim to a standard 10%/90% ^2^H_2_O/H_2_O solution. Samples should ideally be acquired at a constant ambient temperature, i.e., between 18 and 30 °C, and a magnet temperature of 18–30 °C. Shimming to sample optimises resolution within a single minute, and therefore is recommended, but excellent results are achieved with the default shim settings. For the case study reported here, and related LF NMR metabolomics investigations, we routinely performed shimming after each sample analysed in order to attain high-quality spectra, and also to maintain a linewidth below 0.55 Hz for reference samples. Both urine and salivary supernatant samples are dominated by a large water signal at ~4.8 ppm in ^1^H NMR spectra; these require a preliminary 1D proton spectrum to be acquired (~10 s) in order to identify the exact frequency position of this resonance. This intense water signal must be sufficiently suppressed in order to explore the dynamic range of metabolites, and therefore to identify the precise chemical shift value of this signal (δ = ~4.80–5.00 ppm for our LF 60 MHz spectrometer), which is temperature-dependent, and inputting this exact resonance chemical shift value into the water suppression sequence optimises signal-to-noise ratio (SNR) and significantly reduces signal overlap (this process may be conducted automatically within the spectrometer software package). For our experiments conducted at ambient temperature, the optimal water presaturation frequency was found to be 4.95 ppm. Preliminary pilot experiments performed on aqueous glucose calibration standards (100–400 mmol/L) established that the manufacturer’s default setting presaturation power level of 58 dB served as the most efficient for optimising SNR. Values of 62 and 65 dB were less effective, and extremes of 30 and 70 dB were of little or no value. Appropriate repetition times between scans are determined using a *T*_1_ experiment on the sample in order for the optimum relaxation time to be determined (5 * T_1_). SNRs can be improved by increasing the number of scans, scaling with a factor to √2, i.e., quadrupling the number of scans will result in a doubling of the SNR. Spectra were acquired using a one-dimensional presaturation (1D PRESAT) sequence to allow for efficient saturation of the water signal, which minimises pertubances to the remaining signals in spectra acquired. The parameters used for these analyses are as follows: 64 scans, an acquisition time of 6.4 s, a repetition time of 10 s, and a pulse angle of 90°. Carr-Purcell-Meiboom-Gill (CPMG) pulse sequences are recommended for plasma samples. The option is provided for these pulse sequences to be scripted, and therefore full automation is possible.

High-field (HF) spectra of urine samples were also acquired using a Bruker Avance-1 400 MHz NMR (Leicester School of Pharmacy, De Montfort University) spectrometer operating at a frequency of 399.93 MHz. The samples were analysed using the noesygppr1d pulse sequence in order to suppress the water signal (δ = ~4.80 ppm) in the urinary samples with irradiation at the water frequency during the recycle and mixing time delays. The free induction decay (FID) was acquired with 32,000 data points using 128 scans and 2 dummy scans, and 3 µs ^1^H pulses, throughout a sweep width of 4844 Hz (12.1 ppm) and a receiver gain automatically adjusted to each sample. 

For further confirmation of urinary glucose concentration determinations performed at 60 MHz, ^1^H NMR spectra of urine samples diluted extensively with ^2^H_2_O were acquired on a JEOL-ECS-400 spectrometer (University of Loughborough facility), with H_2_O/HOD signal presaturation performed at differing power settings. The final ^2^H_2_O content of these final analytical solutions was 81.8% (*v*/*v*). A sweep width of 13.5 ppm was used with 32K data points and a 2.4 s acquisition time, and a finalised presaturation power level of 62 dB was established for the H_2_O/HOD resonance (4.80 ppm). Sixteen pre-scans were employed prior to the acquisition of 256 full scans in order to obtain an acceptable SNR.

## 4. 60 MHz Spectrometer Biomarker Validation

### Calibration

Specific calibration samples of biomolecules relating to diabetes chemopathology were prepared at various concentrations, including 0.015–400.00 mmol/L (and 1.00 mol/L), in HPLC-grade water. For selected metabolites, concentration ranges of 15–800 µM were typically employed. Samples contained 50 µL of 0.40% (w/v) sodium azide, 50 µL of D_2_O containing 0.05% (*w*/*v*) sodium 3-(trimethylsilyl)[2,2,3,3-d_4_] propionate (TSP), 50 µL of 1.00 M phosphate buffer, and 500 µL of analyte solution. This mixture was then rotamixed and added to newly purchased 5-mm NMR tubes ready for analysis. ^2^H_2_O containing TSP was used for these studies, since sample spectra were acquired on both the 400 and 60 MHz instruments. For the benchtop instrument employed here, ^2^H_2_O is not required as a field-frequency lock solvent. However, since we sought to compare datasets acquired from both 60 and 400 MHz instruments, ^2^H_2_O was added to facilitate this process. TSP is an appropriate internal standard for quantification purposes (∂ = 0.00 ppm); therefore, it does not interfere with other signals present in spectra acquired. Sodium azide is added to suppress bacterial growth during periods of sample preparation and storage. Phosphate buffer is used to maintain a constant pH value in order to avoid pH-mediated signal shifts: pH 7.00 or 7.40 is recommended [28]. The purpose of the calibration curve is to optimise analytical quantification, and also to determine the limit of detection (LOD); this represents a resonance intensity which is 3 times the background noise value, and the limit of quantification (LOQ), which is derived from a ratio of 10 times this noise level, as documented in reference [29]. 

**Figure 1:**
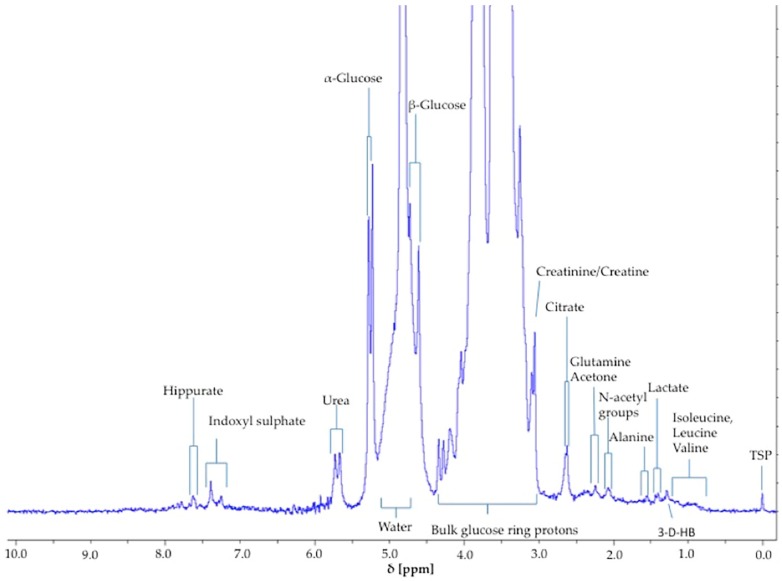
^1^H nuclear magnetic resonance (NMR) type 2 diabetic urinary profile acquired on a 60 MHz benchtop instrument, highlighting a clearly distinguishable β-Glucose-C1-H resonance (*d*, ∂ = 4.65 ppm), in addition to the α-Glucose-C1-H one located at ∂ = 5.25 ppm (*d*), and all further bulk glucose ring structure protons within the 3.19–3.95 ppm chemical shift range for both anomers. Moreover, resonances arsing from a range of further metabolites such as hippurate-CH, indoxyl sulphate-CH, urea-NH_2_, Cn-CH_3_/-CH_2_, creatine-CH_3_/-CH_2_, citrate-CH_2_ (A/B coupling pattern), glutamine-CH_2_, acetoin-CH_3_, acetate-CH_3_, lactate-CH_3_, N-acetyl storage compound-NHCOCH_3_, alanine-CH_3_, isoleucine-CH_3_ and leucine-CH_3_ are also visible in this spectrum. Chemical shifts were referenced to internal tetra-deuterated trimethylsilylpropanoate (TSP) (∂ = 0.00 ppm). Abbreviations: 3-d-HB, 3-d-hydroxybutyrate-CH_3_.

**Figure 2:**
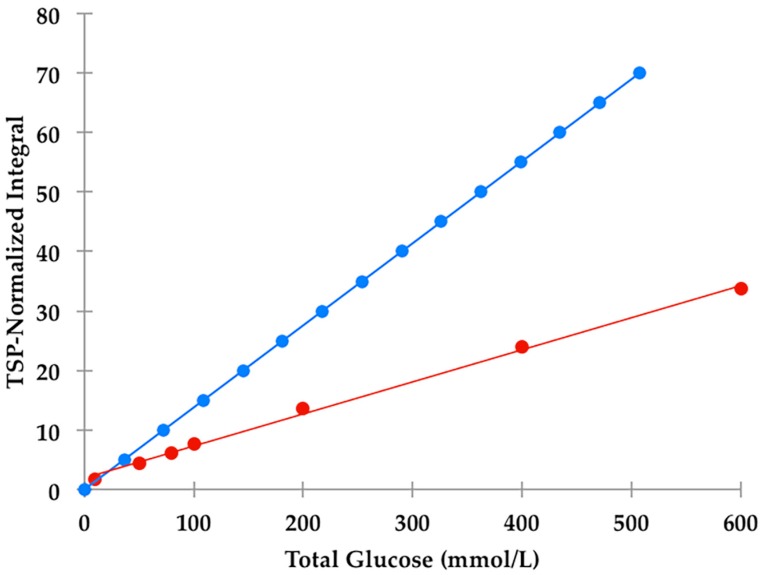
Calibration curve plot of the α-Glucose:TSP (δ = 5.25:0.00 ppm) resonance integral ratio vs. total glucose concentration in phosphate-buffered aqueous solutions (pH 7.00) containing *ca.* 10% (*v*/*v*) ^2^H_2_O (red plot). Glucose concentrations ranged from 10.0–600.0 mmol/L, and that of the TSP internal standard was maintained at a final concentration of 223 µmol/L. The blue plot represents that derived from TSP-normalised integral values predicted directly from the known concentrations of total glucose and TSP present, the relative numbers of ^1^H nuclei contributing towards their ^1^H NMR resonances (1 and 9 respectively), and the 36% abundance of the α-glucose anomer (δ = 5.25 ppm signal).

A series of glucose calibration standard solutions (100–600 mmol/L) were employed to calibrate the 400 MHz Bruker Avance AV-1 HF spectrometer via determinations of the TSP-normalised α-anomeric-C1-H proton resonance (*d*, δ = 5.25 ppm) intensities, and plotting these against concentration. This calibration exercise was performed as described above, and excellent linear relationships between these normalised intensities and glucose standard concentrations were observed (Figure 1 and Figure 2: R^2^ = 0.994). 

Similarly, the 400 MHz JEOL-ECS-400 HF spectrometer was also employed for the above glucose standard solution calibrations. Again, very highly significant linear correlations were found between the TSP-normalised α-anomeric-C1-H glucose proton resonance intensity and total glucose standard concentration (Section 6, R*^2^* = 0.989). 

## 5. 60 MHz ^1^H NMR Benchtop Spectrometer Protocol: Biofluid Analysis Recommendations

### 5.1. Experimental Design and Research Ethics Approval

Prior to sample collection, ethical factors and experimental design should be carefully considered. All samples in our example study were collected with informed consent and approved by the appropriate Research Ethics Committee, specifically the Faculty of Health and Life Sciences Research Ethics Committee, De Montfort University, Leicester, UK (reference no. 1936). All participants were primarily provided with participant information sheets (PISs), and were then required to sign a project consent form in the presence of a researcher witness. The PIS clearly informed those recruited that since their participation was voluntary, they had the freedom to withdraw from the investigation at any stage of its progress. All participants were also requested not to consume any alcoholic beverages, nor any dietary sources known to affect the human metabolome, for 24 h prior to urine sample collection. Essentially, all ethics considerations were in accord with those of the Declaration of Helsinki of 1975 (revised in 1983). 

A pre-established experimental design is crucial prior to commencing sample collection, since factors such as age, sex, body mass index (BMI), fasting, exercise, stress, drug intake and supplements all affect the human metabolome. Samples can be collected over a period of time to monitor the pharmacokinetics and metabolism of drugs, and samples may be collected randomly, or under pre-fasted or non-fasted conditions. Essentially, the fewer lateral variables available, the more precise the study of the metabolome becomes for that particular disease manifestation. Moreover, the appropriate incorporation of demographic variables such as age, gender, body mass index, etc., in such models also enhances precision, but those incorporating relatively large numbers of these require larger or much larger population sample sizes. Validation of specific biomarkers includes the performance of larger trials, and/or repetition of the trial by a different laboratory for validation purposes. Furthermore, it is now quite common for metabolomics researchers to employ a large volume pooled human biofluid mixture (collected from healthy controls or otherwise) to serve as a validated reference sample for all current and future investigations. 

Contradictions in metabolomics experiments may commonly arise from experimental error, caused primarily from the high turnover rate of metabolites in terms of stability, solubility and volatility, together with unacceptable sample preparation techniques. Therefore, experimental design is critical for preparation of the experiment itself. Repeated freeze/thaw cycles should be avoided for all sample types [15], and factors such as maintaining samples at ambient temperature for prolonged periods of time should be kept to a minimum. Buffering procedures should be kept uniform. It is also important to reduce the number of experimental steps, limit sample handling, process samples rapidly, maintain samples at a cold or frozen state prior to analysis, and analyse these as soon as possible thereafter. 

### 5.2. Sample Collection and Sample Storage

Sample preparation can be automated or manually performed. Automation of sample preparation approaches involves a barcoding system which ensures participant anonymity in such studies. Robotic processing of the samples is also possible, allowing for samples to be prepared and analysed in bulk quantities. This increases productivity, but more importantly displays applicability and viability in a point-of-care setting. It is important to define and follow experimental design and Standard Operational Procedures (SOPs) in order to avoid the acquisition of erroneous results. 

#### 5.2.1. Urine

Participants should fast for a ≥12-h period prior to providing a sample, since diet is known to affect the urinary metabolome, for example a higher intake of fruit leads to elevations in rhamnitol, 4-hydroxyhippurate, tartarate, hippurate and glycolate in urine [30]. Urine samples should be collected in sterile, plastic universal containers. Urine specimens should be transported to the laboratory on ice, and then centrifuged immediately (3500 rpm at 4 °C for 15 min). The supernatants are then stored at −80 °C prior to analysis, although temperatures below −25 °C are usually adequate [31].

#### 5.2.2. Saliva

Volunteers should fast for a 12-h period prior to providing a sample, and it is preferable that participants provide samples immediately after awakening in the morning. Participants should refrain from activities such as smoking, eating, drinking, brushing teeth, etc. during the period between waking and sampling, in order to avoid analytical interferences arising from external activities [32,33]. Saliva samples should be collected in sterile, plastic, universal containers, transported to the laboratory on ice, and then centrifuged immediately (3500 rpm for 15 min.) in order to remove cells and debris. The supernatants are then stored at −80 °C prior to analysis. Human salivary supernatant analysis was not included in the type 2 diabetes case study documented below; results from studies featuring the LF 60 MHz NMR analysis of this biofluid will be reported in detail elsewhere. 

#### 5.2.3. Blood Plasma/Serum

Whole blood should be collected following a 12-h fasting period. Blood samples are collected by a fully trained phlebotomist via venepuncture. For plasma, lithium heparin tubes should be used for collection to avoid analytical complications arising from the use of tubes containing ethylenediaminetetra-acetate (EDTA) or citrate anticoagulants. EDTA or citrate present in collection tubes will not only give rise to interfering resonances themselves, but will also chelate metal ions, i.e., Mg^2+^ and Ca^2+^, a process which generates further interfering signals in the ^1^H NMR spectral profiles acquired, i.e., those of the Ca^2+^-EDTA and Mg^2+^-EDTA complexes, which are distinct from those of EDTA itself in view of a slow exchange of this chelator on the NMR timescale. However, often it is preferable to allow the blood to clot and isolate serum samples therefrom via a simple centrifugation step. Samples should be immediately centrifuged at 4 °C, 4300 rpm for 15 min. Serum and plasma samples are then stored at −80 °C prior to analysis. 

### 5.3. Sample Preparation

Biofluid samples are thawed at ambient temperature and then immediately prepared. Preparation for the test set of samples described herein involved centrifuging 500 µL volumes of sample (plasma, serum, urine or saliva) and removing 450 µL of the clear supernatant for analysis. A 50 µL aliquot of phosphate buffer at pH 7.00 (1.00 mol/L) was added to the supernatant, which contained 0.05% (*w*/*v*) sodium azide (prepared in HPLC-grade water), and then 50 µL of ^2^H_2_O, also containing 0.05% (*w*/*v*) TSP (Sigma-Aldrich), was then added to the solution, so that the final (*v*/*v*) content of this deuterated solvent was ca. 10%. The final added TSP concentration for these mixtures was therefore 264 µmol/L. Exceptionally for plasma and serum samples, and also other high protein content biofluids such as knee-joint synovial fluid, the TSP singlet resonance is substantially broadened in view of its binding to proteins therein, and hence is best avoided (although it may be included as a standard solution placed in a capillary insert within an NMR tube). Therefore, only ^2^H_2_O was added in this case. These mixtures were then rotamixed and transferred to newly purchased NMR tubes ready for analysis. 

Where required or specified, differing dilution processes incorporating higher (*v*/*v*) contents of ^2^H_2_O were employed for analysis where specified in the urinary case study outlined below.

### 5.4. Sample Acquisition

Please refer to the recommended acquisition parameters in Section 3. 

### 5.5. Preprocessing

A variety of software modules can be used for the essential preprocessing of bioanalytical ^1^H NMR datasets prior to multivariate metabolomics analysis: Mestranova (Santiago de Compostela, Spain), JEOL Delta (Tokyo, Japan), Bruker Topspin 4.0 (Billerica, MA, USA) and ACD Labs 12.0 (Toronto, ON, Canada). Free induction decays acquired by such instrumentation can be automatically preprocessed, including corrections for linewidths (apodisation), Fourier transformation, phase correction, baseline correction and data alignment. The apodisation/window function is employed in order to smooth minor spectral artefacts arising from the FID process, which surround resonances of interest, to zero. Sidelobes to resonances can also be suppressed, although this process increases linewidths and decreases resolution. Fourier transformation is a mathematical process which converts the FID time functions into frequency ones, which yield the commonly observed format of NMR spectra in which, depending on operating frequency, resolution between all signals observed are optimised in view of their differing chemical shift values. This readily allows comparisons between corresponding 60 and 400 MHz spectra acquired on the same samples. In this format, further preprocessing strategies can be applied in order to optimise the quality of spectra acquired. 

Preferably, NMR signals should be as symmetrical as possible, with a consistent baseline of ~0, and have clear, defined and narrow lineshapes. Baseline and phase corrections can be manipulated manually for more precise corrections, or automatically using predefined coefficient parameters. Baseline correction allows for manipulation of the spectral baseline, adjusting the entire spectrum to start at a set value of 0. Without baseline corrections, data may show a non-uniform signal area when integrated, which is attributable to fluctuations in the baseline and does not arise from the concentration of particular metabolites present in samples investigated. Phase correction allows for the manipulation of the spectrum to alleviate the effect of phase shifting. A poorly adjusted phase correction can result in the absorption signal dipping beneath the spectral baseline, which can lead to an erroneous integration of negative resonance areas. Using a phase correction, the signal region can be manipulated from its negative absorbance into the desired pure absorption signal. Apodisation involves multiplying the FID pointwise by a defined function in order to improve the lineshape within the spectrum. A Gaussian function is commonly used in view of its ability to improve the resolution of signals by narrowing their linewidths; however, this also increases the spectral noise intensity. On occasion, it is prudent to apply a Lorentzian function to improve the SNR, at a cost of broadening the signals and hence reducing resolution and sine bell functions. The weakness of apodisation occurs when applying a correctional function, causing the resonance to broaden; as such, deformation of signals may occur, a process potentially leading to the misidentification of multiplet signals.

### 5.6. ^1^H NMR Metabolite Assignments

TSP acts as a reference signal standard (∂ = 0.00 ppm) for aqueous biofluid analyte samples in order to ensure that metabolites can be chemical shift-aligned accordingly, although alternative or study-specific NMR reference agents, which may also serve as quantitative internal standards, may also be employed. Databases for reference samples include the Human Metabolome Database (HMDB) [13], Madison Metabolomics Consortium Database (MMCD) [34], and COLMAR Metabolomics Web Server [35], which aid metabolite assignments. Further experiments for structural elucidation include the use of two-dimensional (2D) techniques such as correlation spectroscopy COSY and total correlation spectroscopy TOCSY strategies, both of which are available on LF, benchtop instruments. Essentially, 2D techniques can show ^1^H-^1^H, and where appropriate, ^1^H-^13^C correlations using a grid style map so that investigators may readily determine intramolecular connectivities between nuclei of interest therein. Limitations of databases for authentic biomolecule reference compounds include metabolites predominantly being determined at 500–600 MHz operating frequencies, which could lead to problems with interpreting spectral data at LF. However, the use of NMR-SIM and Guided Ideographic Spin System Model Optimisation (GISSMO) software strategies enables structural calculations, i.e., simulations of spins at different magnetic fields, and can also afford valuable information on both individual molecules and relatively complex mixtures [36,37]. Thus, a combination of databases and simulations are the most appropriate approach for structural elucidation strategies using LF instrumentation. 

Computational simulations of the ^1^H NMR spectra of biomolecules such as alanine, glucose, etc., at increasing operating frequencies were performed using ANATOLIA, WinDNMR, NMR-SIM and/or SpinWorks software modules. 

### 5.7. Integration and Data Manipulation

Bucketing (otherwise known as binning) represents the manual, fixed frequency range or ‘intelligent’ computational selection of NMR chemical shift (ppm) frequency zones which selectively contain single ^1^H NMR resonances. However, it should be noted that the required degree of single resonance selectivity is much more limited an operating frequency of 60 MHz than it is at medium to very high fields, i.e., at operating frequencies of 400–800 MHz. Hence, spectral resonances are grouped into either resonance-specific buckets (either manually or computationally, the latter known as ‘intelligent’ bucketing) or evenly spaced windows (fixed frequency range bucketing, e.g., at fixed 0.04 or 0.05 ppm intervals). These are then summed so that the area of each resonance within each spectral ‘window’ represents a cumulative integral. This builds up to the total ^1^H NMR spectral intensity, which represents 100% for constant sum normalisation (CSN) purposes described below.

Manual bucketing requires the user to observe signals and bucket them accordingly so that particular pre-specified resonances are incorporated therein, the objective being that only those resonances are bucketed. Intelligent bucketing is automated and buckets integral datasets according to programming software and the parameters provided, using an algorithm to integrate selected bins. Since ^1^H NMR chemical shift values are critically dependent on temperature, pH and ionic strength, the bucketing step must be carefully performed. Hence, manual integration ensures that only one metabolite signal is incorporated into its corresponding bucket, although there are obviously limitations with what may be achieved from this in LF benchtop NMR profiles. Indeed, it should be noted that at an operating frequency of only 60 MHz, at least some overlap with interfering, lower-intensity signals is virtually inevitable. Uniform binning was not performed, since this can lead to issues such as particular resonances splitting between and the inclusion of >1 signal within these uniformly selected buckets. Buckets were then imported into MS Excel in order to create a ^1^H NMR data matrix, and data normalised to constant sum or to an internal standard reference agent of known added concentration accordingly. Any buckets attributed to baseline noise, water or other interfering agents were eliminated prior to analysis. Normalisation may also be performed by the expression of resonance intensities relative to that of the internal reference (TSP), in order to calculate absolute urinary metabolite concentrations. However, for diabetic urine samples, it was not possible to normalise biomolecule concentrations to those of urinary creatinine (Cn) at a LF benchtop spectrometer operating frequency of 60 MHz, since the high glucose levels therein significantly overlapped with Cn’s characteristic >N-CH_3_ and –CH_2_- function resonances (both singlets, δ = 3.03 and 4.05 ppm respectively; Figure 1 and Figure 8), unlike the ^1^H NMR profiles acquired on 400 MHz facilities (Figure 8). 

Data filtering may also be performed in order to primarily remove variables which are unlikely to be of value to the modelling of ^1^H NMR datasets. For spectra acquired in the case study demonstrated below, the relatively intense residual H_2_O/HOD resonance region (δ = 4.41–5.16 ppm) was removed from all spectra acquired on the LF 60 MHz benchtop spectrometer, as were the 0.08–1.02, 1.61–1.86, 2.42–2.52, 2.71–2.97, 5.37–5.56, 5.86–6.83, 7.09–7.14, 8.02–8.14 and ≥8.53 ppm regions, in which no ^1^H NMR resonances were visible at this operating frequency in all samples investigated.

### 5.8. Reproducibility

To ensure that results obtained are fully reproducible and reliable, within-assay and between-assay run precisions should be assessed. This is usually performed via the computation of their corresponding components of variance in a pre-designed random effects or mixed model analysis of variance/covariance (ANOVA/ANCOVA) experimental design. Indeed, this ensures that metabolites monitored are consistently reported, and avoids any batch-related errors.

### 5.9. Univariate and Multivariate Statistical Analyses

Statistical analysis can be performed using XLSTAT, Metaboanalyst 4.0, ROCCET, MetATT, Statistica, Python and R software packages, amongst others. Data can be analysed in a univariate or multivariate manner, depending on whether one or more metabolites are targeted. Univariately, each metabolite can be compared using standard deviations and standard errors, box-and-whisker plots, Student’s *t*-tests and ANOVA or ANCOVA strategies in order to assess any differences found. The ‘step-down’ Holm model of the Bonferroni correction, or corrections for false discovery rate (FDR) for the univariate analysis of multidimensional datasets should be adhered to. Multivariate data, which are acquired from ^1^H NMR metabolomic studies, and considers many metabolites simultaneously, can be analysed using statistical techniques such as Principal Component Analysis (PCA), Partial Least Squares-Discriminant Analysis (PLS-DA), Orthogonal Partial Least Squares-Discriminant Analysis (OPLS-DA), Self-Organising Maps (SOMs) and/or Correlated Component Regression (CCR) analysis strategies, for example.

PCA is an unsupervised technique which assists with the detection of any statistical ‘outliers’ in datasets, and following their removal, PC clusterings can be explored for each classification group, if indeed there are any distinguishing metabolic features present. However, it is important to note that if any outlier samples are indeed detectable in 2D or 3D PCA scores plots, then a rigorous further analysis of the causes of such outlier datapoints is required, in order to explore any possible explanations for this. Valid reasons for the exclusion of such outlier samples include issues or errors arising from (1) sample collection, storage and/or preparation, and (2) ^1^H NMR spectral acquisition. Moreover, the ^1^H NMR detection and of selected drugs employed for the treatment of the condition under study, and the non-removal of their resonances, together with those of corresponding urinary xenobiotic metabolites, prior to the performance of multivariate metabolomics analysis, represents a further reason, as may any possible co-morbidities or further disorders related to the condition investigated (e.g., hypertension and obesity in type 2 diabetic patients). Similarly, alcohol consumption may also affect the results acquired (via the facile ^1^H NMR detection of urinary ethanol), as may tobacco smoking habits. Indeed, in the case study documented below, a single healthy control urine sample out of a total of 15 samples collected was found to contain resonances ascribable to ethanol, and hence this sample was removed from the dataset prior to statistical analysis.

PCA is also a valuable strategy for the detection of any sub-clusterings of human participants within the same patient disease cohorts, e.g., those perhaps arising from co-morbidities, or demographic variables such as age, gender, BMI, etc. 

Notwithstanding, PLS-DA is a supervised technique which is able to seek and evaluate any significant distinctions between datasets using permutation and further testings. PLS-DA is able to distinguish between metabolites causing the most separation between the datasets, and these are statistically validated and cross-validated in order to confirm that the testing performed is sound. Moreover, the CCR technique has the ability to generate reliable metabolomics predictions from datasets in which the number of correlated explanatory metabolite variables (*P*) is greater or much greater than the sample size (*n*), irrespective of any multicollinearities between these predictor variables.

Validation of these statistical techniques can be performed using the leave-one-out cross-validation (LOOCV) approach, the Q^2^ statistic and area under the receiver operating characteristic (AUROC) curve. AUROC describes the validity of a model based on sensitivity and specificity, i.e., the extent of objects correctly identified within a model, and that of samples correctly classified as ‘foreign’, respectively. A plot of sensitivity vs. (1-specificity), i.e., true positives vs. false positives, is described as a ROC curve, which extends to a multidimensional hypersurface should there be multiple classes considered. The area under the ROC curve (or AUROC value) acts as a measure of class separation, where a value of unity corresponds to optimal separation between two classes of classification, while a value of 0.50 indicates no separation whatsoever [38].

### 5.10. Computational Intelligence (Neural Networks)

Computational intelligence techniques (CITs), including support vector machines (SVMs), optimisation algorithms (genetic algorithms, Ants) and machine-learning algorithms (supervised and unsupervised neural networks), can be employed for the analysis of multivariate (MV) ^1^H NMR datasets acquired in order to recognise metabolic classification patterns for diseases, as well as disease severity indices for each of several biofluids investigated, i.e., as non-probabilistic binary classifiers. Optimisation algorithms can be used to select the most relevant (discriminatory) biomarker variables from datasets for use in constructing a machine-learning classification model. Random forest (RF) techniques can also be employed for classification and variable selection, in which datasets are divided into training and test sets. The performance is assessed using an out-of-the-bag (OOB) error value, and the accuracy, specificity and sensitivity can be obtained from the test set. Self-organising maps (SOMs) can explore self-similarities between spectra and hence clusterings that arise from each source of variation involved. The RF model can be repeated multiple times in order to prevent bias arising from the random sub-sampling of the training and test sets. Unsupervised learning methods can also be employed in order to perform data-mining, and hence uncover any ‘hidden’ patterns.

### 5.11. Pathway Identification

The Kyoto Encyclopedia of Gene and Genomics (KEGG), MetaCyc and HMDB each aim to aid in ascribing significant biomolecular modifications identified to imbalances in or perturbations to established metabolic pathways, and can also identify any linkages between cycles. Upregulation and downregulation of cycles can be analysed using these platforms which enable areas of the body/cell/organism to be assessed metabolically. Metaboanalyst 4.0 also interfaces with these databases. Moreover, the connectivities of biofluid metabolites that potentially distinguish between diseases and corresponding healthy controls, and also disease severity classification groups, can, in principle, be explored through the Metabolomics software module of Ingenuity Pathways Analysis (IPA, Ingenuity Systems, Redwood City, CA, USA). Indeed, canonical pathways analysis can be employed to identify those that are the most significantly different between the above disease or severity group classifications.

## 6. Case Study: Urinary Profiles of Fasted Diabetic Patients with Elevated Glucose Levels

### 6.1. Preliminary calibrations and validation

As an example of the above protocol, a case study was performed for the monitoring of glucose levels in type 2 diabetic urine samples, together with additional metabolites which parallel such a marked upregulation in patients with this condition. Samples were collected and acquired using the above protocols and parameters. The type 2 diabetic (n = 10) and healthy control (n = 14) participants had mean ± SEM ages of 45 ± 3.9 (range 37–76; seven female/one male) and 27 ± 2.7 (range 21–56; nine female/five male) years respectively (a single heathy control sample was excluded in view of the ^1^H NMR detection of ethanol in one of the original 15 samples collected for this classification). All diabetic participants were teetotal and non-smokers, and had a mean ± SEM type 2 diabetes duration of 4.86 ± 2.09 years at the time of sample collection. 

The α-glucose anomer C1-H signal located at ∂ = 5.25 ppm (*d*) (Figure 1) was detected and integrated, and its intensity expressed relative to that of TSP (*s*, δ = 0.00 ppm). This particular resonance was employed for quantification purposes since urinary ^1^H NMR profiles contain many overlapping signals within the crowded 3.00–4.00 ppm range in which glucose’s C2-H to C6-H proton resonances are located, and in view of the poor resolution of these at 60 MHz, this was selected as the optimal resonance to monitor in diabetic and prospectively diabetic patients. Moreover, although distinguishable, the C1-H doublet resonance for β-glucose (δ = 4.65 ppm) was too closely overlapped with the residual water signal for quantification purposes (Figure 1). However, it should be noted that glucose’s α-anomer only represents 36% of the total glucose concentration present (the remaining 64% being the β-anomer), and therefore the factor 100/36% (=2.78) should be employed for converting α-anomer concentrations to total glucose ones. The limit of quantification for total (α-plus β-anomer) glucose was 8.0 mmol/L (section S1, Supplementary Materials section), a value corresponding to 2.88 mmol/L for α-glucose. The calibration curve exhibited a clear linear relationship (r = 0.9973: Figure 2). The limitations of monitoring this α-glucose resonance using LF benchtop NMR analysis is ascribable to potential interference of the closely located residual water signal, together with influence of the continuous water presaturation process on this closely located and spectrally intense resonance centred at δ = 4.95 ppm; this interference is further investigated and reviewed below. Indeed, total concentrations of this key biomarker below 8.0 mmol/L were found to be unquantifiable in view of a significant degree of overlap of the residual presaturated water signal of ≥10% integration intensity contributory interference (observed at added levels of <8.00 mmol/L). Typical spectra acquired on 5.00, 8.00 and 10.00 mmol/L total glucose calibration standards are shown in section S1 of the Supplementary Materials file. The calculated SNR values for the 8.00 mmol/L total glucose standard was 21, and 11 for each method employed in order to estimate this quantity; when the mean of the 10 data points located both sides of the α-glucose-C1-H resonance, and only the 10 largest noise data-points, respectively, were considered. Consideration of lower total glucose concentrations (6.00–7.50 mmol/L) gave rise to a water disturbance (contribution) to the α-glucose-C1-H signal of ≥10%, and therefore 8.00 mmol/L was considered to represent the LOQ value for these LF ^1^H NMR analyses. 

From Figure S1b, the α-glucose-C1-H resonance for glucose present at a total level of 5.00 mmol/L was unobservable at 60 MHz operating frequency, although those of the bulk carbohydrate ring protons (C2-H to C6-H_2_) were.

Of particular bioanalytical and ^1^H NMR-linked metabolomics interest, we noted that there was a major difference between the observed TSP-normalised α-glucose C1-H NMR resonance intensities and those calculated from the known total glucose and added TSP concentrations, the relative numbers of ^1^H nuclei contributing towards their ^1^H NMR resonances employed for analytical purposes (1 and 9 respectively), and the 36% abundance of the α-glucose anomer. Indeed, these ratios were reproducibly only 42% of those predicted from their known total glucose standard concentrations (Figure 2); this effect is clearly visually apparent in the comparative 60 versus 400 MHz spectra shown in Figure 8. This observation provides powerful evidence that the H_2_O/HOD presaturation sequence applied at a power setting of 58 dB (selected from pilot optimisation experiments) substantially diminishes the intensity of this anomeric proton resonance located at δ = 5.25 ppm, and which is only ca. 18 Hz away from the water frequency at an operating frequency of 60 MHz (δ = 4.95 ppm). This frequency difference is much greater at an operating frequency of 400 MHz (ca. 180 Hz), and therefore this presaturation process presumably exerts a much lesser effect on the intensity of this α-anomeric C1-H resonance (although it indeed does so on glucose’s corresponding β-anomeric C1-H signal centred at δ = 4.65 ppm, as outlined in detail below). 

Despite this, provided that all NMR facilities employed for urinary glucose concentrations are carefully calibrated with a set of freshly prepared glucose calibration standard solutions, particularly those analysed at only 60 MHz operating frequency, then such presaturation effects are circumventable.

However, we successfully detected and created calibrations for other biomolecules at significantly lower concentrations through optimised experiments, such as acetone, which we found had a LOQ value of ca. only 25 µmol/L using a 60 MHz benchtop facility (Figure S2, section S2 of the Supplementary Materials section). Typical linear calibration plots of TSP-normalised resonance intensities versus added biomolecule concentrations for acetone (‘spiked’ into heathy control urine samples), betaine and phenylalanine (both in aqueous solution containing ca. 10.0% (*v*/*v*) ^2^H_2_O), are shown in section S3 of the Supplementary Materials document. Moreover, for citrate calibration standards (0–20.0 mmol/L, spectra acquired with both 16 and 64 scans), there was an excellent agreement between the concentration estimated from its TSP-normalised resonance intensity (AB coupling pattern, δ = 2.65 ppm) and its known standard calibration value (r = 0.9914). Moreover, we found that the reproducibility of these measurements made on the 20.0 mmol/L standard was ±0.10 mmol/L).

The α-glucose signal is easily identified in the ^1^H NMR spectral profiles of urine samples collected from non-rigorously controlled type 2 diabetic patients. However, there is a small level of overlap between the water signal and the α-glucose signal, a phenomenon complicating integration and hence quantification of this key biomarker at total concentrations <8.00 mmol/L. Optimisation was attempted by moving the H_2_O/HOD driver signal to ∂ = 4.80 ppm to ensure the best clarity between these two signals; however, there was still a residual level of overlap. Despite these problems, we found that integration of glucose’s α-anomeric proton resonance (δ = 5.25 ppm) was affected negligibly if the urinary concentration of this anomer was ≥2.8 mmol/L (corresponding to a total glucose level of ≥ca. 8 mmol/L). To date, urinary profiles have not been acquired on LF, benchtop NMR systems for metabolomics analysis such as that used in this example. Indeed, additional ^1^H NMR signals in addition to those of glucose and assignable in LF 60 MHz spectra may also be employed for metabolomics analysis, notably ketone bodies which arise from the use of lipid sources as a fuel in patients with poorly controlled diabetes. Such a multivariate metabolomics analysis of our LF 60 MHz ^1^H NMR dataset was therefore performed, and results arising therefrom are outlined below. 

**Figure 3:**
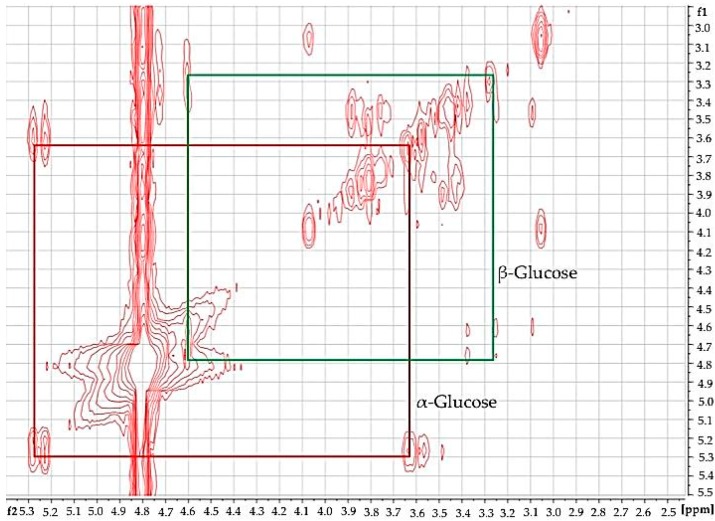
2D ^1^H-^1^H COSY NMR diabetic urinary profile acquired on a 60 MHz instrument highlighting connectivities between α-Glucose-C1-H and -C2-H resonances at ∂ = 5.25 ppm (*d*) and 3.52 ppm (*dd*), respectively (labelled in red), and correspondingly, those of the β-anomer at δ = 4.65 (*d*) and 3.23 ppm (*dd*), respectively (labelled in green. A further ^1^H-^1^H COSY connectivity between signals located at δ = 3.06 and 4.08 ppm is also clearly visible.

Using 2D ^1^H-^1^H COSY analysis (Figure 3), the identity of glucose in diabetic urine samples was readily confirmed, since this revealed connectivities between the -C1-H and -C2-H resonances of both its anomeric forms. Moreover, further glucose and other biomolecule connectivities were visible. This demonstrates the usefulness of 2D ^1^H-^1^H COSY analysis of human urine as a confirmatory tool for LF benchtop NMR-based metabolomics applications.

Primarily, we compared urinary glucose determinations acquired on a LF 60 MHz benchtop NMR system with those conventionally determined on two separate HF 400 MHz facilities. 

When all NMR spectrometer facilities employed for this study were correctly and rigorously calibrated with standard glucose concentrations in accordance with the strategy used for the 60 MHz LF benchtop instrument (i.e., using 0–600 mmol/L total glucose concentration standard solutions), there was an excellent correlation between urinary glucose determinations made on the 60 and both 400 MHz systems (R^2^ ≥ 0.997). Moreover, paired sample *t*-tests found no significant differences between total urinary glucose concentration determinations performed on a LF benchtop 60 MHz spectrometer and the two more conventional HF 400 MHz NMR facilities (*p* = 0.079 and 0.324).

However, further investigations were required in order to explore highly significant deviations from the 36:64 ratio of α-glucose:β-glucose C1-H anomeric proton resonance intensities (i.e., those of the corresponding 5.25:4.65 ppm signals) observed in urinary spectra acquired on both the 400 MHz facilities employed for these studies. In view of this crucial observation, further comparative evaluations between these results and those acquired at 60 MHz were performed. Such studies served to facilitate the direct determination of urinary glucose concentrations on these HF facilities without reference to calibration standard solutions. Indeed, for the ^1^H NMR spectral profiles acquired on type 2 diabetic urine samples at an operating frequency of 400 MHz, we observed that the ratios of intensities of glucose’s α- and β-C1-H anomeric proton resonances was 57:43 (mean ± SEM percentage α-anomer C1-H signal intensity 57.11 ± 2.40%), a value which reproducibly deviated substantially from the expected value, specifically 36:64, i.e., 36% α-anomer (this is also readily visible in the 400 MHz profile shown in Figure 8). This indicated that the H_2_O/HOD presaturation process employed and its corresponding power setting gave rise to a marked ‘dampening’ of the β-anomer’s C1-H NMR signal intensity, which arises from its very close chemical shift locality (δ = 4.65 ppm) to that of the presaturation frequency (δ = 4.80 ppm). In view of its close locality, this resonance is, of course, expected to be more affected by this presaturation process than the α-anomeric one located at δ = 5.25 ppm. Therefore, it appeared that the default NMR power setting of 50 dB employed for H_2_O/HOD solvent suppression at an operating frequency of 400 MHz gave rise to this unexpected and erroneous anomeric ratio. 

Since the 50 dB default value (which is very effective for simple chemical model system samples) exerted a significant effect on glucose’s anomeric proton intensities, we elected to perform further investigations, which involved the alternative 400 MHz ^1^H NMR analysis of type 2 diabetic urine samples diluted extensively with ^2^H_2_O. In this manner, we developed an alternative HF ^1^H NMR method for the determination of urinary glucose concentrations. For this purpose, 60 µL of urine was diluted to a final volume of 0.60 mL with ^2^H_2_O, and to this mixture was added 60 µL of a 0.40% (*w*/*v*) solution of the microbicide sodium azide in 1.00 mol/L phosphate buffer (pH 7.00). These solutions, which contained 81.75% (*v*/*v*) ^2^H_2_O and only 18.25% (*v*/*v*) H_2_O were therefore much more suitable for electronic integration of glucose’s two anomeric proton signals, i.e., the adverse effects of water resonance presaturation were minimised in this high ^2^H_2_O content solution medium. To demonstrate this, we compared the integration ratios of these two anomeric proton resonances (δ = 4.65 and 5.25 ppm for the β- and α-anomers respectively) in the type 2 diabetic urine samples under these low H_2_O content sample preparation conditions, and employing a lower presaturation power setting of 62 dB (only one quarter of that of the 50 dB one), to those acquired on these samples containing only ca. 10% (*v*/*v*) ^2^H_2_O and the 50 dB default power setting provided above. Data acquired demonstrated that the mean relative intensity of the α-C1-H resonance signal to that of total glucose was 37.84 ± 1.96% (n = 6 samples), which is in excellent agreement with the expected value (36.0%). Therefore, this approach was considered highly satisfactory, and it was henceforth employed for all our HF 400 MHz ^1^H NMR glucose determinations on HF spectrometers.

Moreover, these further experiments established that the lower power setting of 56 dB (56 dB equating to one-half the power of the default 50 dB one) also produced integral values much closer to the expected 36:64 ratio; that employing the 62 dB power setting (a further halving of the power) generated a further improved integral ratio value for HF bioanalytical ^1^H NMR glucose determinations. Full results acquired from these studies will be reported elsewhere. 

Having further established the validity of our integration strategy for the two anomeric protons of glucose at an operating frequency of 400 MHz, we then again sought to compare glucose concentrations determined from the TSP-normalised intensities of the α-anomer to those acquired on the LF 60 MHz benchtop spectrometer. We also compared these two sets of urinary total glucose concentrations to those determined on non-NMR analytical methods, i.e., an established glucose oxidase-peroxide/4-aminophenzone/phenol (GOD-PAP)-based spectrophotometric method (outlined in section S4 of the Supplementary Materials), along with a simpler but less accurate urinary glucose visual colourimetric dipstick test system (Health Mate, DUS 8, DFI Co. Ltd., Gyeongsangnam-do, Korea). Where required, urine samples were diluted 1/5 or 1/10 prior to visual colourimetric dipstick analysis. 

For this purpose, we employed an analysis-of-variance (ANOVA)-based experimental design with two main sources of variation (Equation (1), in which M*_i_*, represents that attributable to any differences between the four analytical methods/techniques employed (fixed effect), P*_j_* that arising from differences ‘between-participants’ (random effect, substantial in this case), e*_ijk_* that ascribable to fundamental error, and y*_ijk_* the urinary glucose concentration and μ that concentration in the absence of these sources of variation.

y*_ijk_*= μ + M*_i_* + P*_j_*+ e*_ijk_* (1)

**Figure 4:**
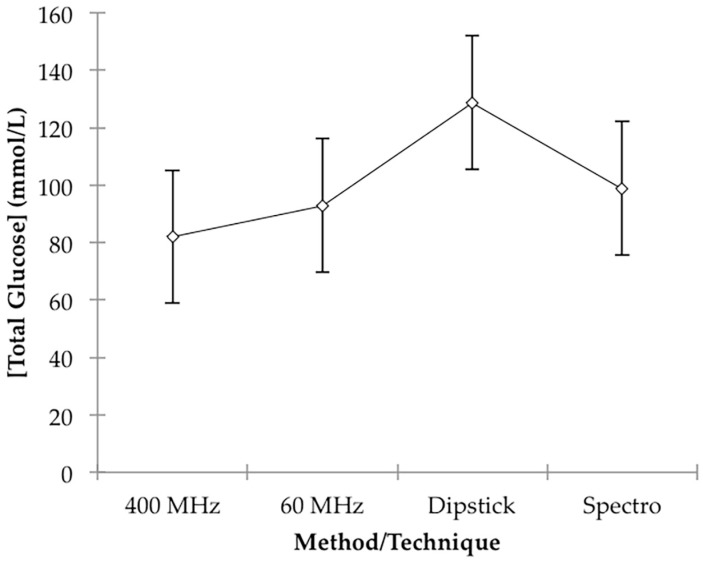
Plots of mean ± 95% CIs urinary glucose concentrations determined from analysis by low field (LF) 60 MHz benchtop ^1^H NMR, conventional HF 400 ^1^H MHz NMR, the GOD-PAP spectrophotometric (abbreviated Spectro) and chromophoric dipstick (abbreviated Dipstick) test systems. The wide confidence intervals are predominantly ascribable to the highly statistically significant ‘Between-Participants’ random effect component-of-variance (P*_j_* in Equation (1)), and not analytical reproducibility.

This analysis found that there were very highly significant differences between participants (*p* < 10^−4^), as expected; however, that between methods/techniques was barely statistically significant, and the only difference found between these four form of analyses using further analysis by Tukey’s highest significant difference (HSD) test, was that between 400 MHz ^1^H NMR-determined glucose levels and those obtained from the above dipstick approach, the latter being significantly greater than the former (*p* = 0.033). However, this is not unexpected in view of the much poorer accuracy of the dipstick colour test system employed. Mean total estimated glucose concentration values for the 60 MHz NMR, 400 MHz NMR, spectrophotonetric GOD-PAP and chromophoric dipstick analyses were 92.9, 82.0, 98.8 and 128.7 mmol/L respectively, and these clearly indicate that the simple, less accurate dipstick analysis system may have overestimated urinary glucose levels. Plots of mean ± 95% ‘Between-Participant’ confidence intervals (CIs) for each class of determinations are shown in Figure 4.

Therefore, based on this ANOVA experimental design, there were no statically significant differences found between our analysis of glucose on a LF (60 MHz) benchtop NMR facility and those determined by HF ^1^H NMR spectroscopy (400 MHz), an established glucose oxidase-based spectrophotometric assay, and also a simpler colour-visual dipstick strategy.

A plot of total glucose level results acquired on the LF 60 MHz NMR facility versus those obtained with the HF 400 MHz one was indeed linear (R^2^ = 0.980), and 95% CIs for the y-intercept and regression coefficient (gradient) covered 0.00 and 1.00, respectively (0.888–1.189 for the latter parameter), information further confirming an excellent agreement between these two bioanalytical ^1^H NMR approaches. However, although plots of our 60 MHz NMR data against those arising from the spectrophotometric and dipstick analyses were again linear (R^2^ = 0.940 and 0.980 respectively), and 95% CIs for the y-intercepts of these plots covered zero, 95% CIs for the regression coefficients were found to be significantly less than the 1.00 value expected for good agreement between these values (0.621–0.943 and 0.648–0.819 respectively). These plots, with 95% CIs for both means and observations, are shown in section S5 of the Supplementary Materials section. Although as noted above, higher total glucose levels were expected for the simple dipstick test, the ca. 20% higher concentration values observed for the spectrophotometric analysis system are not simply explicable, and further investigations are underway to explore this difference further.

Despite excellent linear relationships between them, there were also statistically significant deviations from unity (i.e., <1.00) for the regression coefficients of plots of the 400 MHz analysis results against both the spectrophotometric and dipstick analysis systems explored, although the upper CIs for the former parameter were found to be very close to unity.

### 6.2. ^1^H NMR-Linked Metabolomics Analysis of LF 60 MHz Benchtop Spectrometer Datasets: Type 2 Diabetes Versus Healthy Controls

Both univariate and multivariate analysis of the LF benchtop 60 MHz NMR dataset revealed clear and highly statistically significant differences between urine samples collected from a cohort of diabetic patients (n = 10) and those from healthy controls (n = 14). The dataset acquired, comprising 27 manually selected and electronically integrated bucket regions ranging from 1.03–8.52 ppm, was normalised to the TSP internal standard (of final concentration 264 µmol/L), and then potential predictor variables within chemical shift bucket columns were generalised-logarithmically (glog)-transformed, and Pareto-scaled (Pareto-scaling involves subtraction of the mean resonance integration bucket value from all bucket observations followed by their division by the square root of that variable’s standard deviation, so that each one has a mean value of 0 and a variance not equivalent but similar to unity. Additional analysis performed was conducted exactly as described above, but with constant sum rather than added internal standard (TSP) concentration normalisation CSN. 

Primarily, 27 univariate two-sample Student’s *t*-tests were performed, and when corrected for FDRs, these revealed very highly significant differences in the urinary concentrations of a range of biomolecules in type 2 diabetic patients. Key biomarkers detected using the LF ^1^H NMR technique were: citrate, i.e., 2 × -CH_2_CO_2_^−^ functions within the relatively spectroscopically clear 2.53–2.70 ppm bucket (*p* = 1.87 × 10^−6^); N-acetyl storage compounds, i.e., N-acetylsugar- and N-acetylamino acid-NHCOCH_3_ function protons in the relatively clear 1.99–2.13 ppm bucket (*p* = 1.87 × 10^−6^); lactate as its -CH_3_ group protons in the 1.25–1.34 ppm region (*p* = 2.15 × 10^−6^), which has potential interferences arising from threonine- and acetoin-CH_3_ functions; alanine as its -CH_3_ function doublet resonance at 1.48 ppm with minimal potential interferences; Cn, i.e., as its >N-CH_3_ proton singlet within the 2.98–3.14 ppm bucket (*p* = 5.0 × 10^−6^), with potential interferences arising from creatine-CH_3_, lysine-ε-CH_2_ and γ-aminobutyrate’s γ-CH_2_ functions, and also β-glucose’s C2-H 3.21 ppm signal; acetone as its -CH_3_ groups’ singlet resonance within the 2.14–2.29 ppm bucket (*p* = 5.53 × 10^−6^), which has conceivable interferences from glutamine-C3-CH_2_ and acetoin-CH_3_ proton signals; acetate as its -CH_3_ function in the 1.87–1.99 ppm bucket (*p* = 4.53 × 10^−5^), which has a potential interference from the thymine-CH_3_ resonance, although it should be noted that the latter metabolite has a substantially lower urinary concentration than the former; 3-d-hydroxybutyrate within the 1.14–1.25 ppm bucket (*p* = 4.55 × 10^−5^), with potential interferences arising from 3-aminoisobutyrate-CH_3_ and L-fucose-CH_3_ function resonances; indoxyl sulphate in the 7.15–7.33 ppm bucket (*p* = 6.27 × 10^−3^), with a potential interference from tyrosine’s C2/C6 aromatic proton doublet signal; and hippurate as its signal localised within the 7.55–7.71 ppm bucket, the only potential interfering agent being 1-methylhistidine’s C4 imidazole ring proton singlet (*p* = 0.037). Most importantly, glucose, which was determined firstly as a composite bulk carbohydrate ring proton (i.e., C2-H to C6-H_2_) bucket (δ = 3.14–3.99 ppm, *p* = 2.15 × 10^−6^), and secondly as the more specific α-anomeric proton (i.e., α-C1-H) resonance bucket (δ = 5.17–5.36 ppm, *p* = 0.038) was also found to be a key upregulated biomarker, as expected. However, as noted above, a complication of the α-C1-H glucose signal’s integration is its small fractional overlap with the residual water signal at 60 MHz operating signal. Although the bulk 3.14–3.99 ppm glucose sugar ring proton bucket intensity can be expected to be influenced by contributions from those of a range of further urinary metabolite signals also present within this spectral region, we found that when glucose concentrations were >10 mmol/L, as indeed it was in all six of the type 2 diabetic urine samples explored which had detectable glucose levels (it was non-^1^H NMR-detectable in 4/10 samples investigated), such interferences were limited in view of the much lower intensities of these further biomolecule signals within this broad spectral region (such as those arising from choline, betaine, trimethylamine N-oxide, taurine, glycine, creatine, glycolate, guanadinoacetate, etc.) than those of the relatively intense α- and β-glucose anomers (i.e., C2-H to C6-H_2_ resonances combined).

Upregulations of both the ketone body 3-d-hydroxybutyrate, and also Cn in the type 2 diabetic patient samples were confirmed by HF ^1^H NMR analysis performed at an operating frequency of 400 MHz. Indeed, mean ± SEM absolute urinary concentrations of 3-d-hydroxybutyrate were 3.12 ± 0.99 mmol/L for the type 2 diabetic cohort, but only 0.24 ± 0.06 mmol/L for the healthy control group (*p* = 6.34 × 10^−3^, two-sample Student’s *t*-test), i.e., a 13-fold difference in mean values. Similarly, Cn-normalised 3-hydroxybutyrate levels were 89.8 ± 18.7 and 31.2 ± 6.4 µmol/mmol Cn for the type 2 diabetic and healthy control groups respectively (*p* = 2.86 × 10^−3^). However, TSP-normalised intensities of the urinary Cn-CH_2_- function resonance were found to be much higher in the type 2 diabetic patient group, i.e., mean ± SEM values of 14.37 ± 7.27 (corresponding to 20.86±10.56 mmol/L) versus 4.33 ± 0.68 (corresponding to 6.30±0.99 mmol/L) for the healthy control group (*p* = 0.0144). 

**Figure 5:**
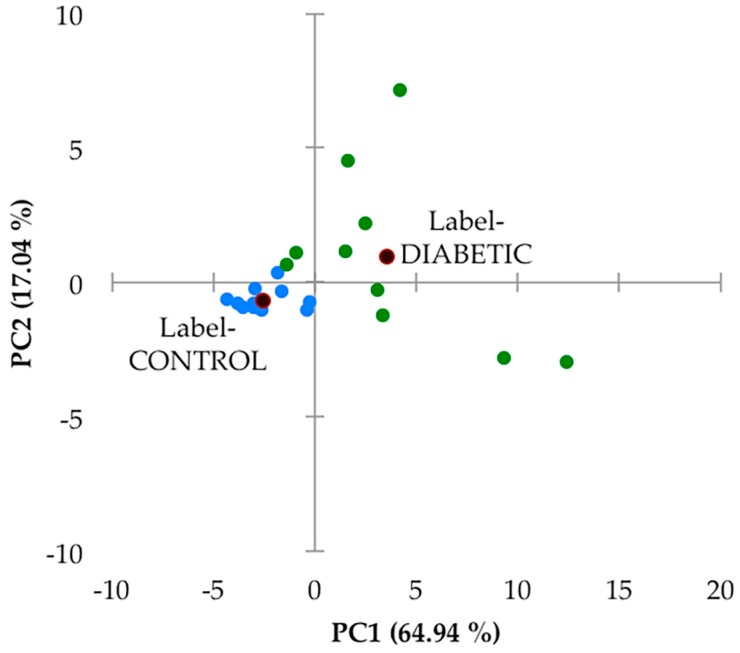
Principal component analysis (PCA) scores plot of PC2 (17.04% of total variance) versus PC1 (64.94% of total variance) for a preliminary investigation of distinctions between healthy control and type 2 diabetic cohorts, and also potential sample outliers. Colour codings: blue, urine samples collected from healthy controls; green, those from type 2 diabetes participants. The black points represent scores plot centroids for the two groups explored. PCA was performed using XLSTAT2014 software, and the dataset was TSP-normalised, generalised logarithmically (glog)-transfomed and Pareto-scaled prior to analysis.

Secondly, this 27-variable dataset was subjected to principal component analysis (PCA), and this analysis, which was performed with varimax rotation and Kaiser normalisation, and a minimum variance criterion of 80%, showed that there were two major PCs isolated, which accounted for a total of 82% of the total model variance. There was a high degree of distinction between the urinary profiles of the two sample groups investigated (Figure 5). However, it should be noted that the PC1 and PC2 scores of samples collected from the type 2 diabetic patient samples varied much more so than their healthy control counterparts. Indeed, PC1 varied from −2 to +13, and PC2 from −3 to +8 for this patient cohort, whereas for the healthy control samples, contributions to both PCs were much lower, and this group of sample PC score datapoints were found to form a tight cluster within the −5 to 0 PC1 range, with only very low PC2 contributions. In view of this high level of PC variation in the type 2 diabetes group samples, we elected not to remove any of the samples as outliers. A detailed manual examination of the ^1^H NMR profiles of both participant groups did not reveal any resonances arising from ethanol, (with the exception of one of the healthy control samples which was removed prior to performing this multivariate data analysis (decreasing the sample size from 15 to 14), and which contained an ethanol-CH_3_ triplet resonance located at δ = 1.19 ppm), nor those from any other xenobiotics. 

An examination of loadings scores revealed that both 60 MHz glucose resonance bucket regions (δ = 3.14–3.99 and 5.17–5.36 ppm) loaded strongly on PC2 (loading scores 0.96 and 0.95 respectively), as did citrate (loading score 0.77), and this accounts for the predominantly higher PC2 values of the type 2 diabetic patient samples (although it should be noted that 4 of these samples had little or no detectable glucose). PC1 had significant loadings from a larger number of these chemical shift bucket regions, including those arising from aromatic biomolecules such as indoxyl sulphate and hippurate, which were found to be downregulated in our type 2 diabetic urinary profiles (as outlined below). Again, PC1 scores were predominantly greater in the diabetic group samples. 

Although no major sub-clusterings of the small number of type 2 diabetic patients recruited to this study (n = 10) were discernable in this PCA scores plot, two urine samples provided therefrom with the most strongly positive PC1 score values (>9 and 12) and PC2 values of ca. −2.9 may be considered as such. However, the small number of samples available clearly restricts any decisions from being made regarding their potential removal. 

**Figure 6:**
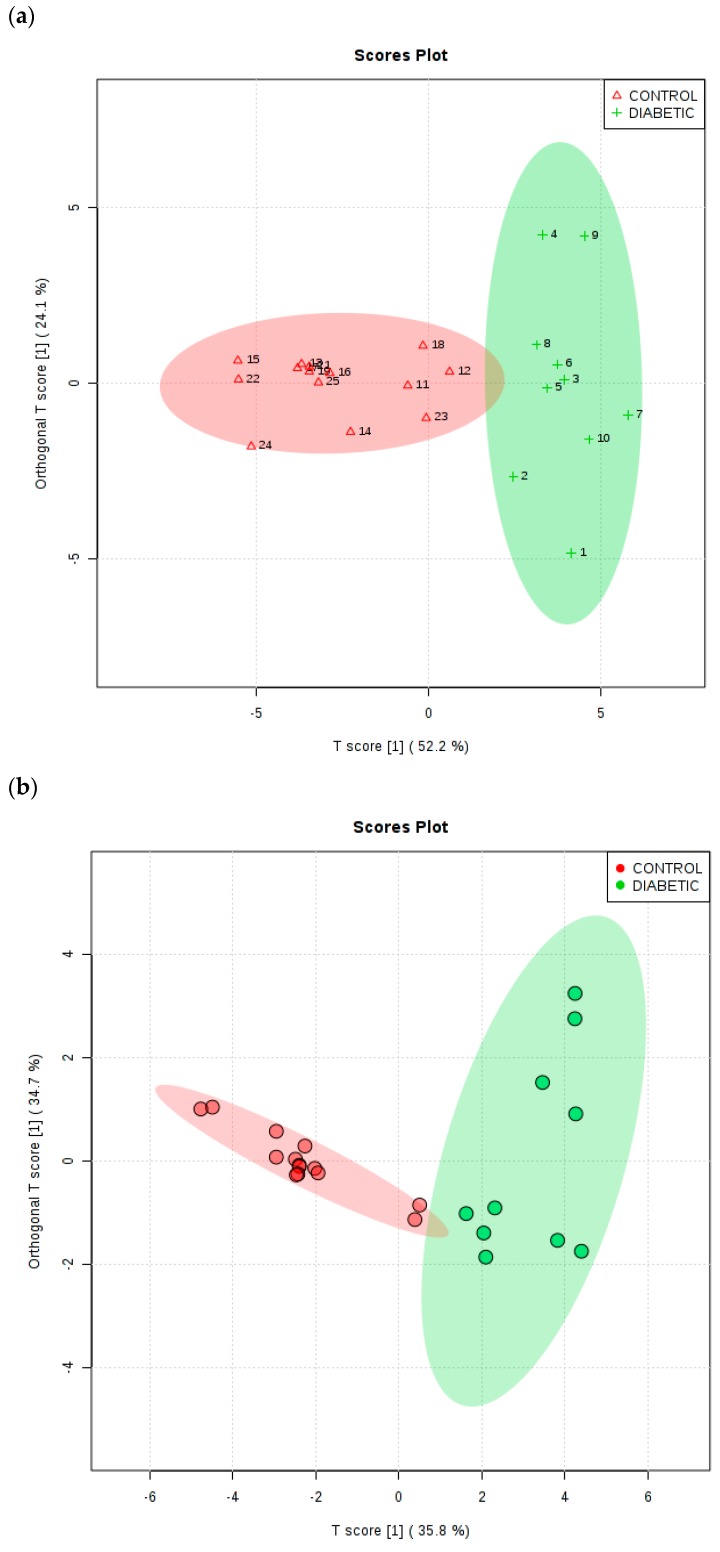
(**a**) Orthogonal projections to latent structures- discriminatory analysis (OPLS-DA) scores plot of orthogonal T score vs. T score for the TSP-normalised dataset demonstrating a clear metabolomics-based distinction between type 2 diabetic patients and healthy controls. 95% confidence ellipsoids are also shown (the type 2 diabetic patient cluster sample T score values (+2.5 to +6) are all greater than those of the control cohort (−6 to +1)); (**b**), as (**a**), but for the constant sum-normalised (CSN) dataset.

As a further example of multivariate analysis, OPLS-DA was utilised to explore the ability of this strategy to distinguish between the type 2 diabetic and healthy control urinary ^1^H NMR profiles acquired. Figure 6a shows an OPLS-DA scores plot with associated 95% confidence ellipses, and this demonstrates clearly distinctive clusterings for these two groups of participants for the TSP-normalised dataset. To evaluate the performance of this multivariate classification system, a 10-fold cross-validation procedure was applied. R^2^X, R^2^Y and Q^2^ values obtained from this analytical model were 0.522, 0.674 and 0.634 respectively, and the Q^2^ value obtained was highly significant (values of this index ≥0.40 are routinely employed as a cut-off for this model, as previously described by Worley and Powers [39]). Moreover, a permutation test conducted with 2000 permutations gave *p* values of <5 × 10^−4^ for both Q^2^ and R^2^Y. PLS-DA variable importance in projection (VIP) scores and OPLS-DA S-plots were utilised in order to identify the most important ^1^H NMR bucket variables for discrimination between healthy and type 2 diabetic participants, and those assigned to methylsuccinate (upregulated in type 2 diabetes) and formate (downregulated) were also found to serve as key biomarker features of this discrimination, in addition to the majority of those detected via the univariate *t*-test analysis described above (including glucose itself, the ketone bodies acetone and 3-d -hydroxybutyrate, acetate, N-acetyl storage compounds, citrate, Cn and lactate).

In view of polyuria experienced by uncontrolled or poorly controlled diabetic patients, we also elected to subject the CSN dataset to MV metabolomics analysis. For this dataset, the OPLS-DA model also yielded a Q^2^ parameter of 0.63 (permutation *p*-value 5.0 × 10^−4^ with 2000 permutations), and the two glucose resonance bucket regions (bulk C2-H to C6-H_2_, and α-anomeric C1-H proton ones) served as the two most important key upregulated biomarker variables for type 2 diabetes (upper right-hand side *p*[1] axis portion of plot, with *p*[1] values ≥ 1.6), as expected. The five most significant type 2 diabetes group-downregulated ones (with *p*[1] values ≤ −2.3 at the lower left-hand side *p*[1] axis of the plot) were exclusively those with relatively intense resonances within the 7.15–8.01 ppm aromatic regions of spectra, i.e., those of indoxyl sulphate and hippurate, together with perhaps lower intensity, spectrally overlapping contributions arising from interfering phenylalanine and 1-methylhistidine signals, respectively. Hippurate’s-α-CH_2_ function resonance (δ = 3.96 ppm) was not among the biomarker resonances found in view of its overlap by much higher intensities of the bulk δ = 3.14–3.99 ppm glucose ring proton signals. As expected, removal of the two glucose resonance bucket regions prior to analysis markedly diminished this model’s Q^2^ value from 0.63 to only 0.43, an observation confirming the importance of these as ^1^H NMR-based distinguishing features.

Similarly, PCA of this glucose resonance-removed CSN dataset showed much more overlap of the type 2 diabetes and healthy control group clusterings than that observed with the inclusion of these signals, and univariate *t*-tests revealed only a small number of significant variables, with FDR-adjusted *p* values ranging from 3.02 × 10^−6^ to 0.032 (these included the alanine and hippurate resonance bucket regions).

**Figure 7:**
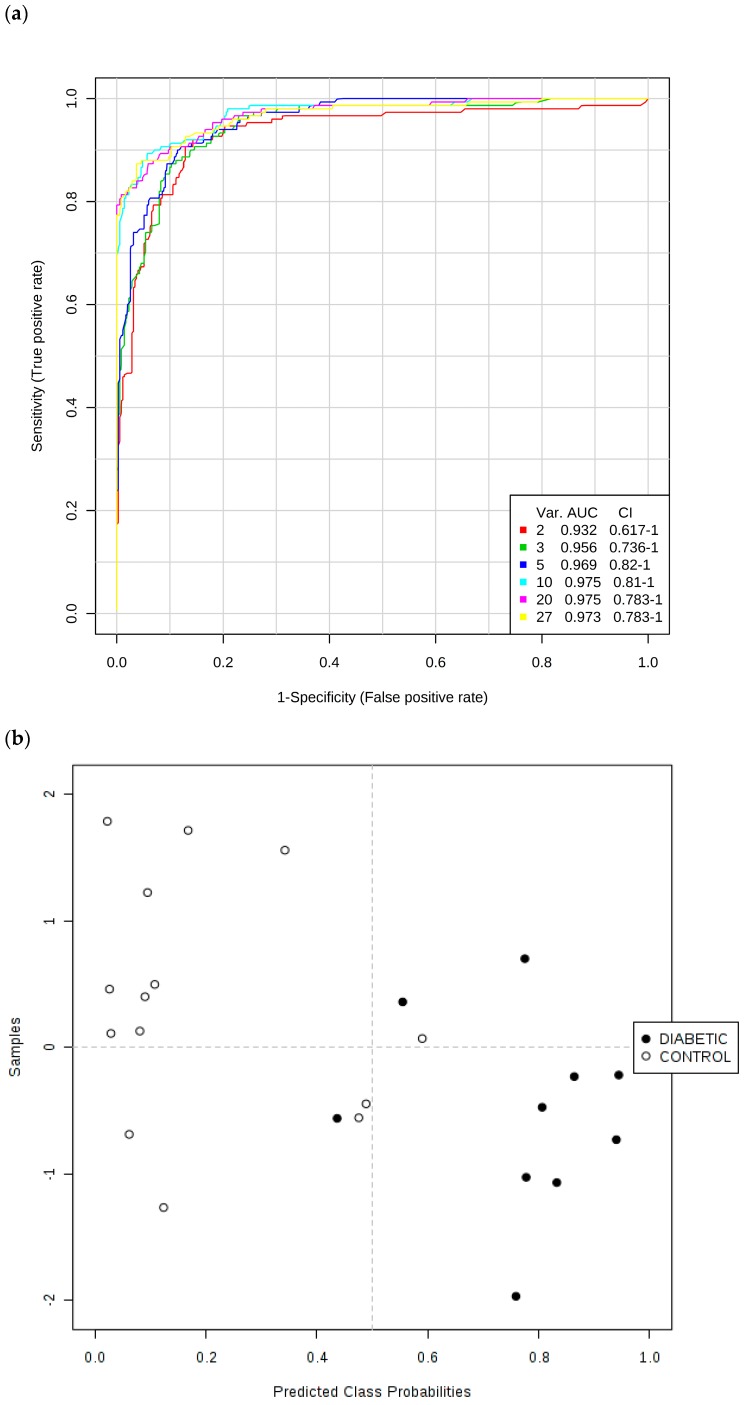
(**a**) Receiver operating characteristic (ROC) curve (plot of true positive vs. false positive rates) with an area under ROC curve (AUROC) value of 0.975 obtained from the support vector machine (SVM) model building system explored with 10 out of a possible 27 variables. ROC curves were developed via Monte Carlo Cross-Validation (MCCV) involving a balanced sub-sampling processes involving application of an SVM model builder (TSP-normalised urinary dataset). The inset shows mean AUROC values estimated for increasing sampling sizes, together with 95% CIs for these values. (**b**) Probability view arising from a balanced sub-sampling approach for SVM model training (predicted class probabilities for each sample employed the most effective area under ROC curve (AUROC)-based classification system).

Secondly, ROC curves produced via Monte Carlo Cross-Validation (MCCV) and based on the SVM strategy demonstrated that the overall mean classification success rate was 97.5% for this model. The most effective SVM models were those which incorporated the total number of 10 chemical shift bucket region intensity features, the AUROC value obtained being 0.975 (95% confidence intervals 0.81–1.00), as shown in Figure 7. Therefore, with the above overall classification reliability and AUROC values, this model applied served as one with a highly effective discriminatory ability (these values are considered effective, highly discriminatory and exceptional for models when they are >0.70, 0.87–0.90 and >0.90 respectively [40]).

Key discriminatory biomarker variables identified from this form of multivariate analysis were citrate > 3-d-hydroxybutyrate > hippurate > N-acetyl storage compounds > alanine > total bulk glucose (C2-H to C6-H_2_ resonances only) > lactate > α-glucose (C1-H resonance only) > 3-(3-hydroxyphenyl)-3-hydroxypropanoate (C1/C6-CH resonances) > indoxyl sulphate > urea in that order of effectiveness.

We also performed this ROC testing system for a simple model incorporating the two TSP-normalised glucose ^1^H NMR buckets, along with those of the ketone bodies acetone and 3-d-hydroxybutyrate, and found that AUROC (and corresponding 95% confidence interval) values for models with two, three and four of these key variables were as high as 0.934 (0.725–1.00), 0.937 (0.773–1.00) and 0.930 (0.773–1.00) respectively, observations confirming that these upregulated diabetes biomarkers were highly significant discriminators, as expected. Moreover, univariate ROC values obtained for a series of key biomarker resonances were found to be 1.00 (N-acetyl storage compounds); 0.9929 (glucose bulk C2-H to C6-H_2_, acetone-CO-CH_3_ and citrate-CH_2_-CO_2_^−^); 0.9430 (3-d-hydroxybutyrate-CH_3_); and 0.882 (α-glucose-C1-H).

Finally, a Random Forest analysis performed on the TSP-normalised dataset with 500 trees and seven distinguishing variables per node successfully classified nine out of ten type 2 diabetic samples, and 12 out of 14 healthy control ones, on the basis of their urinary ^1^H NMR metabolic profiles, i.e., an overall classification accuracy of 0.875 (section S6, Supplementary Materials document).

**Figure 8:**
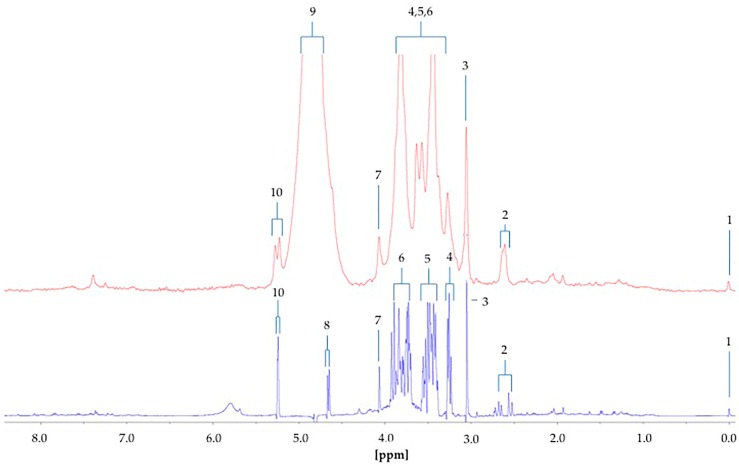
Diabetic urinary ^1^H NMR profile acquired at 60 (red) and 400 MHz (blue) operating frequencies. Assignments: 1, TSP-Si(CH_3_)_3_; 2, Citrate-A/B-CH_2_CO_2_^−^; 3, Cn/Creatine > N-CH_3_; 4, β-Glucose-C2-H; 5, α- and β-Glucose-C4-H/C5-H, and α-Glucose-C2-H; 6, α- and β-Glucose-C3-H/C5-H/C6-H_2_; 7, Cn-CH_2_; 8, β-Glucose-C1-H; 9, H_2_O/HOD-OH; 10, α-Glucose-C1-H.

The difference between the ^1^H NMR metabolic profiles of urine acquired at 60 and 400 MHz operating frequencies for metabolomics analysis is substantial (Figure 8). Indeed, in 1D single-pulse spectra acquired at 60 MHz, the α-glucose signal is influenced by the residual water signal at concentrations <2.8 mmol/L, and increasingly so with decreasing α-glucose level. Moreover, the difference in resolution from 400 MHz to 60 MHz causes significant expansion of multiplet resonances, especially complex second-order ones, at the lower operating frequency. This, in turn, gives rise to resonance overlap problems at 60 MHz, particularly for complex biofluid spectra. Indeed, this resonance δ value expansion would be 400/60 times greater for 60 MHz spectra than it is for 400 MHz ones. For example, the ethanol-CH_3_ function triplet resonance located at δ = 1.19 ppm, which has a *J* value of 7.07 Hz, would wholly encompass 2 × 7.07 Hz/60 Hz = 0.235 ppm of the spectral profile at 60 MHz, but only 2 × 7.07 Hz/400 Hz = 0.035 ppm at 400 MHz. The more challenging signals to assign include those from higher-order multiplets and more highly split first-order ones, such as the lactate C-H quartet resonance (δ = 4.13 ppm). Glucose was clearly ^1^H NMR-detectable in six out of a total of n = 10 type 2 diabetic urine samples at an operating frequency of 60 MHz, and integration of the 5.25 ppm α-anomeric proton signal followed by its normalisation to internal TSP provided calibration curve-based estimates of total urinary glucose levels in our cohort of type 2 diabetic patients. Mean ± SEM glucose concentrations determined by LF benchtop 60 MHz ^1^H NMR analysis were 93 ± 41 mmol/L for all n = 10 samples explored. However, exclusion of the n = 4 type 2 diabetic urine samples in which glucose was undetectable gave revised mean ± SEM values of 155 ± 56 mmol/L. The former mean ± SEM values for Cn-normalised urinary total glucose concentrations determined from our 400 MHz ^1^H NMR analysis were 17.9 ± 7.5 mmol/mmol Cn. These Cn-normalised urinary glucose level values concord with those previously reported in diabetic patients, as noted in Table 1, although it should be noted that these data correspond to type 1 diabetes or diabetic ketosis. This table also lists mean Cn-normalised glucose concentrations for healthy control subjects, which vary from 9.0 to 37.5 µmol/mmol Cn for adults, 31.6 µmol/mmol Cn for children, 7.0–143.1 µmol/mmol Cn for infants, and 15.0 µmol/mmol Cn for new-borns.

The significance of urinary glucose levels is not only evident in diabetes patients, but also in patients with conditions such as eosinophilic esophagitis and Fanconi Bickel Syndrome (Table 1). Therefore, potential applications for using LF-benchtop NMR reach far beyond screening for one disease, metabolic ‘fingerprints’ being able to be formed to successfully diagnose multiple diseases. Moreover, monitoring urinary glucose levels is just a single example of what can be achieved using LF NMR analysis. Investigations of other biofluids and corresponding metabolites are possible, and therefore an abundance of metabolic disturbances may be explored with this novel technique.

## 7. Discussion

While metabolite analysis in intact biofluids by LF NMR spectroscopy is a newly advanced technique, it has the potential to impact on ‘point-of-care’ clinical chemistry diagnosis and monitoring, especially with the increasingly rapid development of spectrometers with operating frequencies >60 MHz. In comparison, HF NMR analysis at operating frequencies of 400–800 MHz has become a standard probing tool for the multicomponent analysis of complex biofluids collected from humans and other organisms. Indeed, a variety of important biological information regarding the molecular nature and concentrations of a wide range of endogenous biomolecules, together with exogenous agents present in such fluids, can be obtained from these investigations. Moreover, biomedical NMR analysis serves as a virtually non-invasive technique, since it often has little or no requirement for the pre-treatment of samples, and generally requires only a limited knowledge of sample composition prior to analysis. These approaches offer significant potential regarding the investigation of metabolic processes, and can be coupled with multidimensional data analysis techniques within metabolomics workflows, serving as an extremely powerful means of probing, for example, the biochemical basis of human disease aetiology. These investigations can also provide substantial diagnostic and/or prognostic disease monitoring information, including the identification and validation of reliable biomarker molecules. This combined NMR-based metabolomics approach is also readily applicable to the simultaneous analysis of a wide range of metabolites in tissue biopsies and cultured cells (either intact via ^1^H high-resolution magic angle spinning (MAS)-NMR-based metabolic profiling strategies, or correspondingly as appropriate solution-state extracts), and/or cell culture media.

Here, we have reported for the first time the rapid, essentially or completely non-invasive analysis of a human biofluid sample (human urine) by a LF 60 MHz benchtop NMR facility. While compact, LF NMR analyses of human plasma, serum, and whole blood can reveal trends in disease development and prognosis through inspection of longitudinal (*T*_1_) and transverse (*T*_2_) relaxation times [51], the major purpose of these pilot studies reported herein was to (a) establish whether 60 MHz benchtop ^1^H NMR spectra may be reliably employed to detect urinary biomolecules, and to determine their concentrations in this biofluid for future diagnostic and prognostic monitoring purposes, despite some inherent resonance overlap phenomena arising from the lower operating frequency involved, and (b) provide a protocol ‘blueprint’ for future NMR-based metabolomics strategies available for the analysis of biofluids. Since the resonance frequencies of nuclei are dependent on the static magnetic field strength, resonances of the same linewidth (in Hz) will appear increasingly broader at decreasing field strengths when expressed relative to the overall spectral ‘window’ range in ppm; as noted here, this gives rise to an enhancement of resonance overlap, particularly in spectra obtained from complex multianalyte samples such as biofluids and tissue biopsy extracts.

Interestingly, the results acquired in this study are at least partially consistent with those obtained by Salek et al., who found that in humans, citrate, Cn/creatine, acetate, 3-d-hydroxybutyrate, and alanine were significantly upregulated in type 2 diabetic urine samples over those of healthy control subjects (^1^H NMR spectral profiles were constant sum-normalised in this investigation) [52]. Moreover, this study removed all glucose resonances prior to MV metabolomics analysis, and one reason for the high discriminatory significance of the type 2 diabetes versus healthy control afforded in the present investigation was ascribable to our inclusion of the two glucose resonance bucket regions in our corresponding analyses.

**Figure 9:**
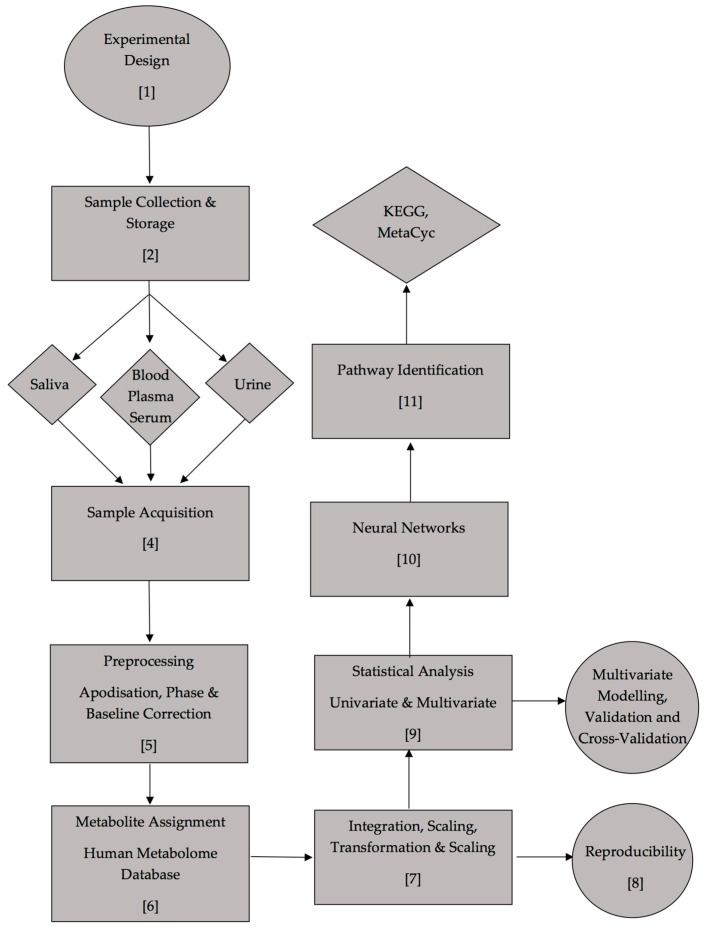
Proposed schematic representation of biofluid analysis by LF benchtop NMR spectroscopy. Numbers refer to sections of the protocol in Part 5 of this work, relevant to each respective section of the scheme. KEGG: Kyoto Encyclopedia of Gene and Genomics

From the protocols and case study described here, a schematic representation has been proposed (Figure 9) in order to allow researchers to follow a protocol for the LF benchtop NMR spectrometroscopic analysis of biofluids, and which is combined with MV metabolomics analysis techniques. This aims to provide full considerations for metabolomics investigations to improve uniformity across the field when using LF benchtop NMR analysis, in order to ensure that some level of protocol uniformity is maintained.

Pilot partial single-pulse LF 60 MHz ^1^H NMR spectra acquired on a healthy human blood plasma sample, which demonstrates the identification of three clearly resolved sets of bulk glucose ring C2-H to C6-H_2_ resonances (as observed in the corresponding 60 MHz urinary profiles shown in Figure 8), is displayed in section S7 of the Supplementary Materials section. The remainder of the these single-pulse profiles were dominated by intense lipoprotein-associated triacyglycerol fatty acid [δ = 0.90 and 1.22 ppm for their terminal-CH_3_ and bulk chain-(-CH_2_-)_n_ functions], and acute-phase glycoprotein molecularly mobile carbohydrate side-chain N-acetylsugar (acetamido -NHCOCH_3_ group) signals (δ = 2.04 ppm), in addition to those arising from a range of protein amino acid residue resonances (both aliphatic and aromatic).

## 8. Limitations of the Pilot Study Performed

The major purpose of the pilot study focused on metabolic monitoring in type 2 diabetic urine samples was primarily to determine whether 60 MHz benchtop ^1^H NMR analytical strategies may be reliably employed to detect urinary biomarkers and further biomolecules, and to determine their concentrations in this biofluid for future diagnostic and prognostic monitoring purposes in patients with this condition, despite some inherent resonance overlap problems encountered at this operating frequency, which is of course much lower than those of more conventional HF spectrometers equipped with large superconducting magnets (e.g., those of 400–800 MHz operating frequencies). Such resonance overlap phenomena present some major analytical problems, especially in spectra acquired on complex multianalyte samples such as human urine. Therefore, some caution should be applied in LF benchtop NMR facility-based investigations involving the quantification of biomolecules and xenobiotics in biofluids, and such issues increase markedly with resonances of higher first-order multiplicities (e.g., quartets and above), or those with higher-order coupling patterns, which may severely limit intensity determinations. However, such interference problems are considered minimal for the determination of major urinary metabolites, i.e., those with prominent resonances in the spectra acquired (e.g., acetate, citrate, alanine, etc.), particularly those with only a minimal level of overlap with lower intensity ones, and also those present in relatively ‘spectroscopically clear’ regions of the profiles obtained, for example urea. Moreover, in the pilot study presented here, the very high urinary concentrations of glucose detectable in type 2 diabetes patients reported here rendered its analysis and determination at this operating frequency relatively facile, despite the high multiplicities and coupling orders, and hence complex coupling patterns of five of its ^1^H NMR signals for both anomeric forms. In addition, quantification of other corresponding highly upregulated biomarkers of diabetes, such as the ketone body 3-d-hydroxybutyrate arising from the NADH-mediated metabolic reduction of acetoacetate (a process catalysed by hepatic β-hydroxybutyrate dehydrogenase), was also readily achievable. Hence, although the 60 MHz ^1^H NMR profiles of human urine are largely dominated by the highest intensity, low coupling pattern and order resonances therein, and/or those assigned to biomolecules of the highest urinary concentrations, this technique also has the ability to provide valuable quantitative, as well as qualitative, information regarding those present at lower or much lower concentrations, including the ketone body acetone which was measurable at levels as low as 25 µmol/L (Figure S2).

There are three further potential limitations of the study performed. Firstly, polyuria associated with poorly controlled or uncontrolled type 2 diabetes gives rise to major reductions in the absolute concentrations of possible biomarkers, and therefore correspondingly proportional TSP-normalised ^1^H NMR resonance intensities, and this may facilitate the achievement of high levels of distinction between this participant classification group and that of healthy controls. Therefore, it should be recommended that all ^1^H NMR-detectable urinary metabolite concentrations should be normalised to that of creatinine as an internal urinary volume marker in order to compensate for this. However, although readily achievable for HF NMR datasets, this form of normalisation was not possible at an operating frequency of only 60 MHz, since there appeared to be at least some significant resonance overlap problems for creatinine’s -CH_2_- function signal (δ = 4.05 ppm) noted in spectra acquired therefrom. However, this LF form of ^1^H NMR analysis, along with that performed here at HF (400 MHz), demonstrated that urinary creatinine excretion was significantly greater in our type 2 diabetes group over that of healthy controls, which further complicates this issue (this observation has been confirmed in humans by Salek et al. [52]). However, a further option is CSN of bucketed urinary ^1^H NMR datasets prior to performing MV metabolomics analysis, preferably following the removal of the highly significant contributions of known upregulated metabolites such as glucose [52], and possibly also those of selected pathologically, but not metabolically, correlating ketone bodies. Indeed, MV metabolomics analysis of the CSN dataset by an OPLS-DA strategy performed here ratified the discriminatory importance of the two glucose resonance bucket regions as biomarker variables for type 2 diabetes, as expected. Moreover, removal of these two glucose bucket regions prior to analysis reduced this model’s Q^2^ value from 0.63 to only 0.43. However, type 2 diabetes-downregulated biomarkers detected from this approach, i.e., aromatic biomolecules such as hippurate and indoxyl sulphate, may arise from diabetic polyuria, as noted above.

Furthermore, univariate ROC values determined for key ^1^H NMR biomarker resonances were extremely high (>0.99) for glucose bulk C2-H to C6-H_2_ resonances, and those of acetone-CH_3_’s and citrate-CH_2_CO_2_^−^’s); very high (>0.94) for 3-d-hydroxybutyrate’s-CH_3_ function signal, and high (>0.88) for the α-glucose-C1-H resonance. Additionally, PCA of this 2× glucose resonance-removed CSN dataset confirmed the prime importance of glucose as a key biomarker when included in the TSP-normalised dataset.

Secondly, as noted above, an additional major explanation for the high level of predictive success of our MV and computational intelligence technique (CIT) metabolomics models applied here is simply the incorporation of predictor metabolic variables, which are known to be primary key up- or downregulated ones involved in the condition or disease evaluated, e.g., upregulated glucose and ketone bodies in type 2 diabetes. Therefore, their removal from the dataset prior to statistical or CIT analyses may serve to facilitate the detection of any secondary metabolic disturbances present, although this will, of course, prevent the further investigation of any MV inter-relationships or correlations between any secondary biomarkers featured therein and the key primary metabolic variables such as glucose.

Thirdly, a further major limitation of ^1^H NMR-based metabolomics studies involving such LF NMR instruments is the possible and sometimes substantial intensity-diminishing effects of the H_2_O/HOD signal presaturation process observed, especially for resonances located close to its frequency (ca. δ = 4.8 ppm), which unfortunately includes the spectroscopically accessible C1-H resonance of α-glucose present in urine samples collected from uncontrolled or poorly controlled diabetics. However, as we have shown, the employment of rigorous calibration processes with biomolecule standard solutions serves to overcome this problem, as does the use of alternative resonances arising from the biomolecule examined, i.e., those which are located at frequencies which are sufficiently remote from the presaturation secondary irradiation one.

Intriguingly, we also observed similar effects of this presaturation process on the relative intensities of both of glucose’s anomeric-C1H resonances at an operating frequency of 400 MHz, and found that an alternative ^2^H_2_O-rich sample preparation strategy, combined with the careful selection and optimisation or presaturation power level, yielded excellent bioanalytical results for urinary glucose determinations in type 2 diabetic patients, which were in very good agreement with those acquired from LF 60 MHz benchtop NMR analysis employing a rigorous calibration standard solution approach.

Interestingly, the effective and robust suppression of strong solvent resonances has attracted much interest within the NMR and NMR-based metabolomics communities for some time [53], and the most suitable approaches are critically dependent on samples analysed, sample pH values and the molecular sizes of analytes considered. However, since such presaturation strategies give rise to a major loss of quantitative information regarding resonances which are localised at similar frequencies to that of the solvent, as noted in the present study, the WET180 sequence has been shown to be the most effective for aqueous solution media [54]. However, it should be noted that in many metabolomics investigations, the application of simple presaturation techniques, in addition to the basic 1D NOESY approach, is still routine.

Finally, an additional complication is ascribable to differences between intramolecular ^1^H relaxation times for particular biomolecular analytes, and also long-range coupling effects. For example, we found that the observed LF 60 MHz spectrum of the amino acid alanine differed substantially from those simulated at that operating frequency from computational analysis performed by ANATOLIA, WinDNMR, NMR-SIM and/or SpinWorks software modules (which provide very accurate chemical shift and coupling constant values). In ^2^H_2_O solution, this amino acid has markedly differing T_1_ relaxation times for its -CH_3_ and α-CH function protons (T_1_ = 1.51 and 6.94 s respectively, as determined in this study), and hence standard ^1^H NMR acquisition parameters (ca. 3 s for both FID acquisition and relaxation delay) can give rise to a deviance in the experimentally observed electronic bucket intensity values from the expected 1:3 ratio for this biomolecule, with that of the α-CH proton being much lower than that predicted. Indeed, the latter was barely detectable or undetectable at calibration standard solutions of <2.0 mmol/L in our experiments (data not shown). Additionally, the effects exerted by a short T_2_ value can give rise to a significant line-broadening effect. Therefore, intramolecular and between-field strength differences in both T_1_ and T_2_ parameters can significantly alter the appearance of selected ^1^H NMR profiles, and currently these influences remain unclear for biomolecules present in complex, multicomponent biofluid mixtures such as human urine. The dependence of these T_1_ and T_2_ values on field strength are significantly different, and decreasing the operating frequency from 400 to 60 MHz is anticipated to decrease T_1_ values by approximately one-half, although only minor modifications to T_2_ are expected.

This further consideration lends further support to the authors’ above recommendation that for future bioanalytical investigations, researchers should focus on the more prominent resonances present in LF NMR biofluid spectra with relatively simple first-order coupling patterns (i.e., singlets, doublets, triplets, etc.) for quantification purposes, with the exception of those in which the targeted analyte is present at relatively high concentrations, for example glucose in diabetic urine samples as reported here. Future developments in LF benchtop NMR technology may indeed provide effective solutions to these problems.

## 9. Conclusions

Biomarkers can serve as powerful predictive indicators of the presence of metabolic diseases, together with a myriad of other conditions, and here we have presented, for the first time, the potential applications of LF benchtop ^1^H NMR analysis to the rapid diagnosis of type 2 diabetes in human urine samples. Therefore, this technique acts as a sensitive means of monitoring the urinary metabolic status of diabetes at ‘point-of-care’ sites, either type 1, type 2 or pre-diabetic conditions, together with gestational diabetes. In view of the very large, inconvenient sizes of HF NMR instruments, hospitals and hospital site clinical chemistry/chemical pathology laboratories certainly do not use these for the rapid, routine and multicomponent analysis for the diagnosis and detection of diseases, but instead may employ such small size LF instruments, rendering them suitable for the clinical chemistry analysis of patient samples directly ‘on-site’ at healthcare settings. Herein, we have demonstrated the detection and quantification capabilities of LF 60 MHz, H_2_O-suppressed spectra acquired on such an NMR instrument, which is based on a rare earth magnet arranged within a Halbach array. In addition to its small size and portability, advantages of the technique regarding the diagnostic and prognostic screening of biofluids for selected human diseases include (1) no requirement for expensive deuterated NMR solvents for sample preparation purposes for some spectrometers available, which (2) most importantly, benefits from ease-of-use, with no requirement for specialist operational staff, and which can, at least in principle, detect very low concentrations of biomolecules present in intact biofluids within short timescales (less than 15 min). The applications of this technique continue to expand, and will likely develop further in the field of disease diagnosis and severity monitoring screening.

## 10. Future Perspectives

LF benchtop NMR has potential to be used as a ‘point-of-care’ diagnostic and prognostic screening facility, if suitable protocols and the validation of biomarker analytes as outlined here are adhered to in clinical practices. The technique is advantageous in view of the low running costs and lack of cryogens required when compared to those of higher resolution, HF facilities commonly found in bioanalytical laboratories. Scripting and robotic sampling can ensure full automation of sample preparation and acquisition, which could later be linked to artificial intelligence techniques.

LF benchtop NMR facilities are becoming more sophisticated, enabling multiple solvent suppression regimens on samples which have been extracted in different solvent systems, for example. This would be particularly applicable to extractions using methanol or chloroform from water-based samples subsequent to a lyophilisation procedure. Furthermore, gradients, which are now available on benchtop NMR systems, extend the availability of such techniques for further methodologies to be developed, such as pure shift, which can assist in the confirmation of signal assignments.

Further NMR-active nuclei such as ^13^C can also be readily monitored in both simple and complex sample matrices using LF benchtop NMR facilities. However, at present, sensitivity for such nuclei is poor, although LOD values for ^19^F are reasonably high. Using techniques such as hyperpolarisation could increase the sensitivity of such nuclei, and may be employed adjunctly with this technology.

Internal standard in instrument ERETIC (Electronic Reference To access In Vivo Concentrations) has emerged to remove the addition of internal standard reference agents completely, and reliance on external references such as TSP may become routine in the near future. Issues with internal reference standards may arise due to their volatility (as for tetramethylsilane, TMS) and potential to bind to macromolecules in high protein content biofluids such as blood plasma/serum, factors which represent major analytical/bioanalytical disadvantages. The field of application for these LF NMR techniques is substantial, ranging from food technology through to ‘at crime scene site’ forensics, and now biomedical imaging, in addition to spectroscopy. While miniaturisation of technology has led to improvements in the analytical performance of these facilities, improvements in sensitivity, and the field strength of the permanent rare earth magnets featured in LF benchtop NMR spectrometers, will lead to further advances in the range of applications of these instruments, and their effectiveness therein.

## Figures and Tables

**Table 1:** Previously reported Cn-normalised urinary glucose concentrations in healthy controls and patients with diabetes, eosinophilic esophagitis and Fanconi Bickel syndrome (results acquired from this investigation are also included). * Error bars unspecified. These data were obtained from the *Human Metabolome Database* (HMDB) [13].

Disease	Age Group	Gender	Mean ± Error * Creatinine (Cn)-Normalised Concentration	Range	Reference
Healthy Control	Adult (>18 years old) healthy control	M/F	36.6 µmol/mmol Cn	10.3–56.7 µmol/mmol Cn	[41]
Healthy Control	Newborns (0–30 days old)	M/F	15.0 µmol/mmol Cn	0.0–50.0 µmol/mmol Cn	[42]
Healthy Control	Adult (>18 years old) healthy control	M/F	9.0 µmol/mmol Cn	0.0–19.0 µmol/mmol Cn	[42]
Healthy Control	Adult (>18 years old) healthy control	M/F	Unavailable	16.7–111.1 µmol/mmol Cn	[43]
Healthy Control	Adult (>18 years old) healthy control	M/F	37.5 µmol/mmol Cn (error bars unavailable)	12.5–58.4 µmol/mmol Cn	[44]
Healthy Control	Adult (>18 years old) healthy control Male	M	31.1 µmol/mmol Cn	Unavailable	[45]
Healthy Control	Infant (0–1 year old)	Unspecified	7.0 µmol/mmol Cn	0.0–15.0 µmol/mmol Cn	[42]
Healthy Control	Adult (>18 years old) healthy control	M/F	25.8 ± 13.8 µmol/mmol Cn	Unavailable	[42]
Healthy Control	Infant (0–1 year old) Female	F	143.1 ± 399.8 µmol/mmol Cn	Unavailable	[46]
Healthy Control	Children (1–13 years old)	Unspecified	31.6 ± 16.0 µmol/mmol Cn	Unavailable	[13]
Diabetes	Adult (>18 years old)	M/F	19.7 mmol/mmol Cn (error bars unavailable)	Unavailable	[47]
Diabetic Ketosis	Adult (>18 years old)	M/F	79.6 mmol/mmol Cn	68.3–90.9 mmol/mmol Cn	[48]
Type 1 Diabetes	Adult (>18 years old)	M/F	17.5 mmol/mmol Cn	0.1–129.9 mmol/mmol Cn	[49]
Type 2 Diabetes	Adult (>18 years old)	M/F	17.9 ± 7.5 mmol/mmol Cn	0–41.93 mmol/mmol Cn	This study
Eosinophilic Esophagitis	Children (1–13 years old)	Unspecified	30.0 ± 27.8 µmol/mmol Cn	Unavailable	[13]
Fanconi Bickel Syndrome	Children (1–13 years old)	F	5.55 mmol/mmol Cn (error bars unavailable)	Unavailable	[50]

## Supplementary Materials

The following are available online at http://www.mdpi.com/2571-5135/8/1/2/s1, Section S1: LF 60 MHz benchtop ^1^H NMR spectra of 5.00, 8.00 and 10.00 mmol./L total glucose calibration standards, Section S2: LF 60 MHz benchtop ^1^H NMR spectrum of a 25 µmol./L acetone claibration standard, Section S3: LF 60 MHz ^1^H NMR calibration standard curves for selected urinary biomolecules, Section S4: Spectrophotometric GOD-PAP determinations of urinary glucose concentrations, Section S5: Plots of urinary glucose concentrations determined by LF 60 MHz ^1^H NMR analysis versus those determined by (a) HF 400 MHz ^1^H NMR analysis; (b) the spectrophotometric GOD-PAP method; and (c) a simple visual colourimetric dipstick approach, Section S6: Random Forest-Metabolomics Classification of the ^1^H NMR Profiles of Type 2 Diabetic and Healthy Control Participants. Section S7: LF 60 MHz single-pulse ^1^H NMR spectral profile of human blood plasma.

## Abbreviations

AI	Artificial Intelligence
ANCOVA	Analysis of Covariance
ANOVA	Analysis of Variance
AUROC	Area under the receiver operating characteristic curve
CCR	Correlated Component Regression
CITs	Computational intelligence techniques
CIs	Confidence intervals
Cn	creatinine
COSY	Correlation Spectroscopy
EDTA	Ethylenediaminetetraacetic acid
ERETIC	Electronic Reference To access In Vivo Concentrations
FID	Free Induction Decay
GISSMO	Guided Ideographic Spin System Model Optimisation
GOD-PAP	Glucose oxidase-peroxide/4-aminophenazone/phenol
HF	High-field
HMDB	Human Metabolome Database
HPLC	High-Performance Liquid Chromatography
IPA	Ingenuity Pathways Analysis
KEGG	Kyoto Encyclopaedia of Gene and Genomics
LF	Low-field
LOD	Limit of Detection
LOOCV	Leave one out Cross validation
LOQ	Limit of Quantification
MAS	Magic Angle Spinning
MCCV	Monte Carlo Cross Validation
MMCD	Madison metabolomics consortium database
MV	Multivariate
NMR	Nuclear Magnetic Resonance
OOB	Out-of-the-bag
PCA	Principal Component Analysis
PLS-DA	Partial Least Squares-Discriminant Analysis
SNR	signal-to-noise ratio
SOMs	Self-Organising Maps
SOP	Standard Operating Procedures
SVM	Support Vector Machine
TCOSY	Total Correlation Spectroscopy
TMS	Tetramethylsilane
TSP	sodium 3-(trimethylsilyl)[2,2,3,3-d_4_] propionate
3-d-HB	3-d-Hydroxybutyrate.

## References

[1] Teng Q. (2013). NMR-Based Metabolomics. Structural Biology: Practical NMR Applications.

[2] Shen B., Tang H., Jiang X. (2016). Translational Biomedical Informatics.

[3] Wishart D.S. (2008). Quantitative metabolomics using NMR. TrAC Trends Anal. Chem..

[4] Santorio S. (1614). De Statica Medicina.

[5] Thomson J.J. (1913). Bakerian Lecture—Rays of positive electricity. Proc. R. Soc. Lond. A.

[6] Purcell E.M., Pound R.V., Bloembergen N. (1946). Nuclear magnetic resonance absorption in hydrogen gas. Phys. Rev..

[7] Pauling L., Robinson A.B., Teranishi R., Cary P. (1971). Quantitative Analysis of Urine Vapor and Breath by Gas-Liquid Partition Chromatography. Proc. Natl. Acad. Sci. USA.

[8] Nicholson J.K., Buckingham M.J., Sadler P.J. (1983). High resolution ^1^H N.M.R. studies of vertebrate blood and plasma. Biochem. J..

[9] Bell J.D., Brown J.C., Nicholson J.K., Sadler P.J. (1987). Assignment of resonances for ‘acute-phase’glycoproteins in high resolution proton NMR spectra of human blood plasma. FEBS Lett..

[10] Logemann J., Schell J., Willmitzer L. (1987). Improved method for the isolation of RNA from plant tissues. Anal. Biochem..

[11] Percival B., Wann A., Masania J., Sinclair J., Sullo N., Grootveld M. (2018). Detection and determination of methanol and further potential toxins in human saliva collected from cigarette smokers: A ^1^H NMR investigation. JSM Biotechnol. Biomed. Eng..

[12] Visentin S., Crotti S., Donazzolo E., D’Aronco S., Nitti D., Cosmi E., Agostini M. (2017). Medium chain fatty acids in intrauterine growth restricted and small for gestational age pregnancies. Metabolomics.

[13] Wishart D.S., Feunang Y.D., Marcu A., Guo A.C., Liang K., Vázquez-Fresno R., Sajed T., Johnson D., Li C., Karu N. (2018). HMDB 4.0: The human metabolome database for 2018. Nucleic Acids Res..

[14] Chong J., Soufan O., Li C., Caraus I., Li S., Bourque G., Wishart D.S., Xia J. (2018). MetaboAnalyst 4.0: Towards more transparent and integrative metabolomics analysis. Nucleic Acids Res..

[15] Trivedi D.K., Hollywood K.A., Goodacre R. (2017). Metabolomics for the masses: The future of metabolomics in a personalized world. New Horiz. Transl. Med..

[16] Blümler P., Casanova F. (2015). Chapter 5. Hardware Developments: Halbach Magnet Arrays. Mobile NMR and MRI.

[17] Qiu Y., Rajagopalan D., Connor S.C., Damian D., Zhu L., Handzel A., Hu G., Amanullah A., Bao S., Woody N. (2008). Multivariate classification analysis of metabolomic data for candidate biomarker discovery in type 2 diabetes mellitus. Metabolomics.

[18] Weljie A.M., Newton J., Mercier P., Carlson E., Slupsky C.M. (2006). Targeted profiling: Quantitative analysis of 1H NMR metabolomics data. Anal. Chem..

[19] Blekherman G., Laubenbacher R., Cortes D.F., Mendes P., Torti F.M., Akman S., Torti S.V., Shulaev V. (2011). Bioinformatics tools for cancer metabolomics. Metabolomics.

[20] Beckonert O., Keun H.C., Ebbels T.M.D., Bundy J., Holmes E., Lindon J.C., Nicholson J.K. (2007). Metabolic profiling, metabolomic and metabonomic procedures for NMR spectroscopy of urine, plasma, serum and tissue extracts. Nat. Protoc..

[21] Blümich B., Casanova F., Dabrowski M., Danieli E., Evertz L., Haber A., Van Landeghem M., Haber-Pohlmeier S., Olaru A., Perlo J. (2011). Small-scale instrumentation for nuclear magnetic resonance of porous media. New J. Phys..

[22] Gouilleux B., Charrier B., Akoka S., Giraudeau P. (2017). Gradient-based solvent suppression methods on a benchtop spectrometer. Magn. Reson. Chem..

[23] Danieli E., Perlo J., Blümich B., Casanova F. (2010). Small magnets for portable NMR spectrometers. Angew. Chem. Int. Ed..

[24] Schaeler K., Roos M., Micke P., Golitsyn Y., Seidlitz A., Thurn-Albrecht T., Schneider H., Hempel G., Saalwaechter K. (2015). Basic principles of static proton low-resolution spin diffusion NMR in nanophase-separated materials with mobility contrast. Solid State Nucl. Magn. Reson..

[25] Singh K., Blümich B. (2017). Desktop NMR for structure elucidation and identification of strychnine adulteration. Analyst.

[26] Masania J., Grootveld M., Wilson P.B. (2017). Teaching analytical chemistry to pharmacy students: A combined, iterative approach. J. Chem. Educ..

[27] Chang W.H., Chen J.H., Hwang L.P. (2006). Single-sided mobile NMR with a Halbach magnet. Magn. Reson. Imaging.

[28] Mickiewicz B., Vogel H.J., Wong H.R., Winston B.W. (2013). Metabolomics as a novel approach for early diagnosis of pediatric septic shock and its mortality. Am. J. Respir. Crit. Care Med..

[29] Armbruster D.A., Pry T. (2008). Limit of blank, limit of detection and limit of quantitation. Clin. Biochem..

[30] Garcia-Perez I., Posma J.M., Gibson R., Chambers E.S., Hansen T.H., Vestergaard H., Hansen T., Beckmann M., Pedersen O., Elliott P. (2017). Objective assessment of dietary patterns by use of metabolic phenotyping: A randomised, controlled, crossover trial. Lancet Diabetes Endocrinol..

[31] Lauridsen M., Hansen S.H., Jaroszewski J.W., Cornett C. (2007). Human urine as test material in ^1^H NMR-based metabonomics: Recommendations for sample preparation and storage. Anal. Chem..

[32] Grootveld M., Silwood C.J.L. (2005). ^1^H NMR analysis as a diagnostic probe for human saliva. Biochem. Biophys. Res. Commun..

[33] Yin P., Lehmann R., Xu G. (2015). Effects of pre-analytical processes on blood samples used in metabolomics studies. Anal. Bioanal. Chem..

[34] Cui Q., Lewis I.A., Hegeman A.D., Anderson M.E., Li J., Schulte C.F., Westler W.M., Eghbalnia H.R., Sussman M.R., Markley J.L. (2008). Metabolite identification via the Madison Metabolomics Consortium Database. Nat. Biotechnol..

[35] Robinette S.L., Zhang F., Brüschweiler-Li L., Brüschweiler R. (2008). Web server based complex mixture analysis by NMR. Anal. Chem..

[36] Dashti H., Westler W.M., Tonelli M., Wedell J.R., Markley J.L., Eghbalnia H.R. (2017). Spin system modeling of Nuclear Magnetic Resonance spectra for applications in metabolomics and small molecule screening. Anal. Chem..

[37] Dashti H., Wedell J.R., Westler W.M., Tonelli M., Aceti D., Amarasinghe G.K., Markley J.L., Eghbalnia H.R. (2018). Applications of parametrized NMR spin systems of small molecules. Anal. Chem..

[38] Lamanna R. (2013). Proton NMR profiling of food samples. Annu. Rep. NMR Spectrosc..

[39] Worley B., Halouska S., Powers R. (2013). Utilities for quantifying separation in PCA/PLS-DA scores plots. Anal. Biochem..

[40] Hanley J.A., McNeil B.J. (1982). The meaning and use of the area under a receiver operating characteristic (ROC) curve. Radiology.

[41] Bouatra S., Aziat F., Mandal R., Guo A.C., Wilson M.R., Knox C., Bjorndahl T.C., Krishnamurthy R., Saleem F., Liu P. (2013). The human urine metabolome. PLoS ONE.

[42] Lentner C. (1981). CIBA-GEIGY Limited. Geigy Scientific Tables.

[43] Putman D.F. (1971). Composition and Concentrative Properties of Human Urine.

[44] Guy P.A., Tavazzi I., Bruce S.J., Ramadan Z., Kochhar S. (2008). Global metabolic profiling analysis on human urine by UPLC–TOFMS: Issues and method validation in nutritional metabolomics. J. Chromatogr. B.

[45] Shaykhutdinov R.A., MacInnis G.D., Dowlatabadi R., Weljie A.M., Vogel H.J. (2009). Quantitative analysis of metabolite concentrations in human urine samples using ^13^C{^1^H} NMR spectroscopy. Metabolomics.

[46] Shoemaker J.D., Elliott W.H. (1991). Automated screening of urine samples for carbohydrates, organic and amino acids after treatment with urease. J. Chromatogr..

[47] Nicholson J.K., O’Flynn M.P., Sadler P.J., Macleod A.F., Juul S.M., Sönksen P.H. (1984). Proton-nuclear-magnetic-resonance studies of serum, plasma and urine from fasting normal and diabetic subjects. Biochem. J..

[48] Hoppel C.L., Genuth S.M. (1982). Urinary excretion of acetylcarnitine during human diabetic and fasting ketosis. Am. J. Physiol. Metab..

[49] Ştefan L.I., Nicolescu A., Popa S., Mota M., Kovacs E., Deleanu C. (2010). ^1^H-NMR URINE metabolic profiling in type 1 diabetes mellitus. Rev. Roum. Chim..

[50] Gupta N., Nambam B., Weinstein D.A., Shoemaker L.R. (2016). Late diagnosis of Fanconi-Bickel syndrome. J. Inborn Errors Metab. Screen..

[51] Cistola D.P., Robinson M.D. (2016). Compact NMR relaxometry of human blood and blood components. TrAC Trends Anal. Chem..

[52] Salek R.M., Maguire M.L., Bentley E., Rubtsov D.V., Hough T., Cheeseman M., Nunez D., Sweatman B.C., Haselden J.N., Cox R.D. (2007). A metabolomic comparison of urinary changes in type 2 diabetes in mouse, rat, and human. Physiol. Genom..

[53] Nguyen B.D., Meng X., Donovan K.J., Shaka A.J. (2007). SOGGY: solvent-optimised double gradient spectroscopy for water suppression. A comparison with some existing techniques. J Magn Reson..

[54] Mo H., Raftery D.J. (2008). Improved residual water suppression: WET180. Biomol. NMR..

