# Supplementary material for: Low-Field, Benchtop NMR Spectroscopy as a Potential Tool for Point-of-Care Diagnostics of Metabolic Conditions: Validation, Protocols and Computational Models
Source: High Throughput. 2018 Dec 27;8(1):2. doi: 10.3390/ht8010002 (PMC6480726; doi:10.3390/ht8010002)
Supplement: Supplementary file 1 [file high-throughput-08-00002-s001.pdf]

Supplementary Materials

Low-Field, Benchtop NMR Spectroscopy as a Potential Tool for Point-of-Care Diagnostics of Metabolic Conditions: Validation, Protocols and Computational Models

Benita C. Percival[1], Martin Grootveld[1], Miles Gibson[1], Yasan Osman[1], Marco Molinari[2], Fereshteh Jafari[1], Tarsem Sahota[1], Mark Martin[3], Federico Casanova[4], Melissa L. Mather[5], Mark Edgar[6], Jinit Masania[1] and Philippe B. Wilson[1,*]

1 Leicester School of Pharmacy, De Montfort University, The Gateway, Leicester, LE1 9BH, UK
2 University of Huddersfield, Queensgate, Huddersfield, HD1 3DH, UK
3 Greater Manchester NHS Trust, Stepping Hill Hospital, Poplar Grove, Hazel Grove, Stockport, SK2 7JE, UK
4 Magritek GmbH, Philipsstraße 8, 52068, Aachen, Germany
5 University of Nottingham, University Park, Nottingham, NG7 2RD
6 University of Loughborough, Epinal Way, Loughborough, LE11 3TU, UK
* Correspondence: philippe.wilson@dmu.ac.uk; Tel.: +44-1162577631

Received: 4 October 2018; Accepted: 13 December 2018; Published: 27 December 2018

SUPPLEMENTARY MATERIALS SECTION

SECTION S1. LF 60 MHz benchtop ^{1}H NMR spectra of 5.00, 8.00 and 10.00 mmol./L total glucose calibration standards.

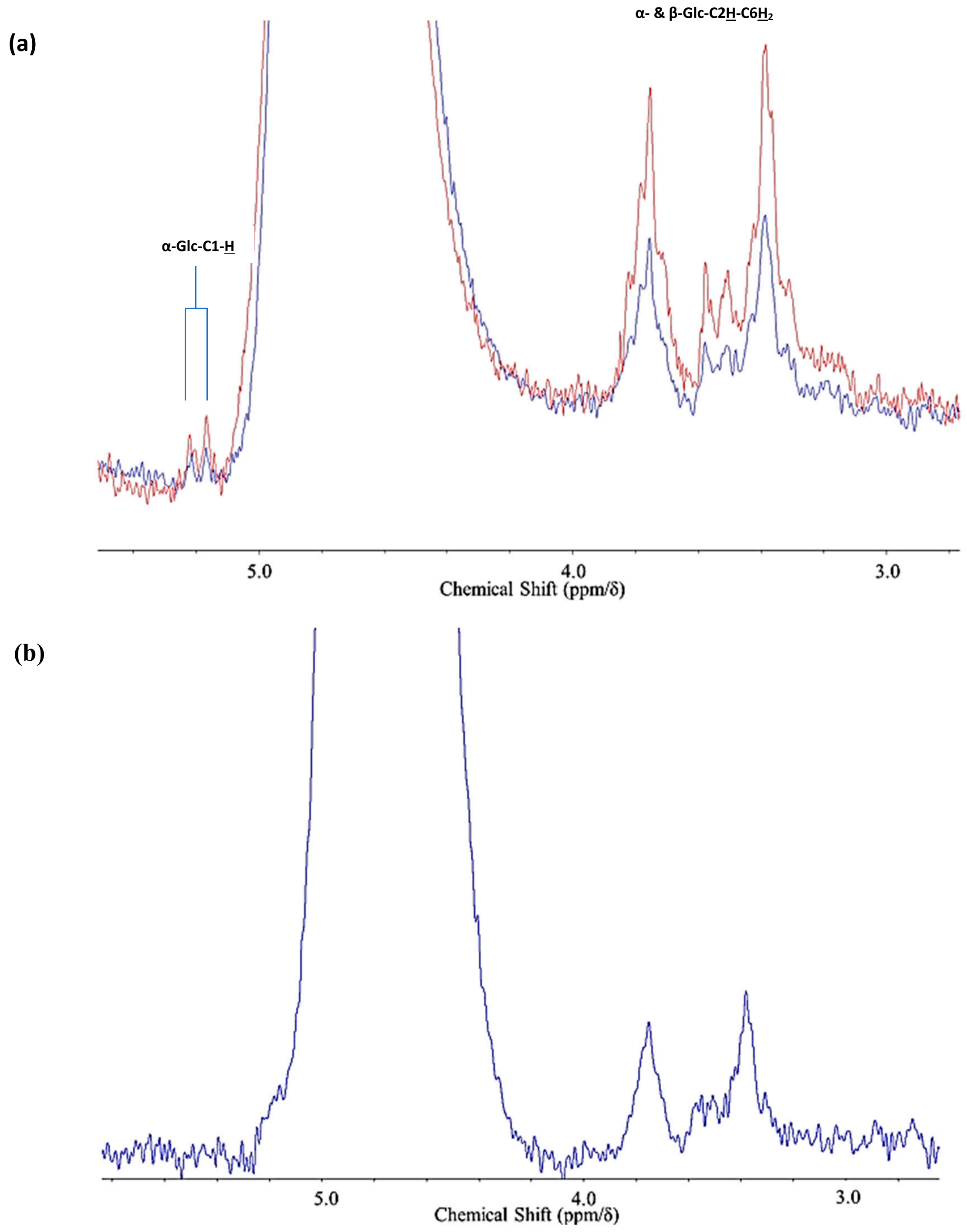

Figure S1. LF 60 MHz benchtop ^{1}H NMR spectra of **(a)** 10.00 and 8.00 mmol./L total glucose (red and blue respectively); and **(b)** 5.00 mmol./L total glucose. Typical spectra are shown. Abbreviations: *α*-Glc-C1-H, alpha-Glucose-C1-H; *α*- & β-Glc-C2H-C6H$_2$, *α*- and β-Glucose bulk carbohydrate ring-C2H to -C6H$_2$ protons. Typical spectra are shown.

SECTION S2: LF 60 MHz benchtop ^{1}H NMR spectrum of a 25 µmol./L acetone claibration standard

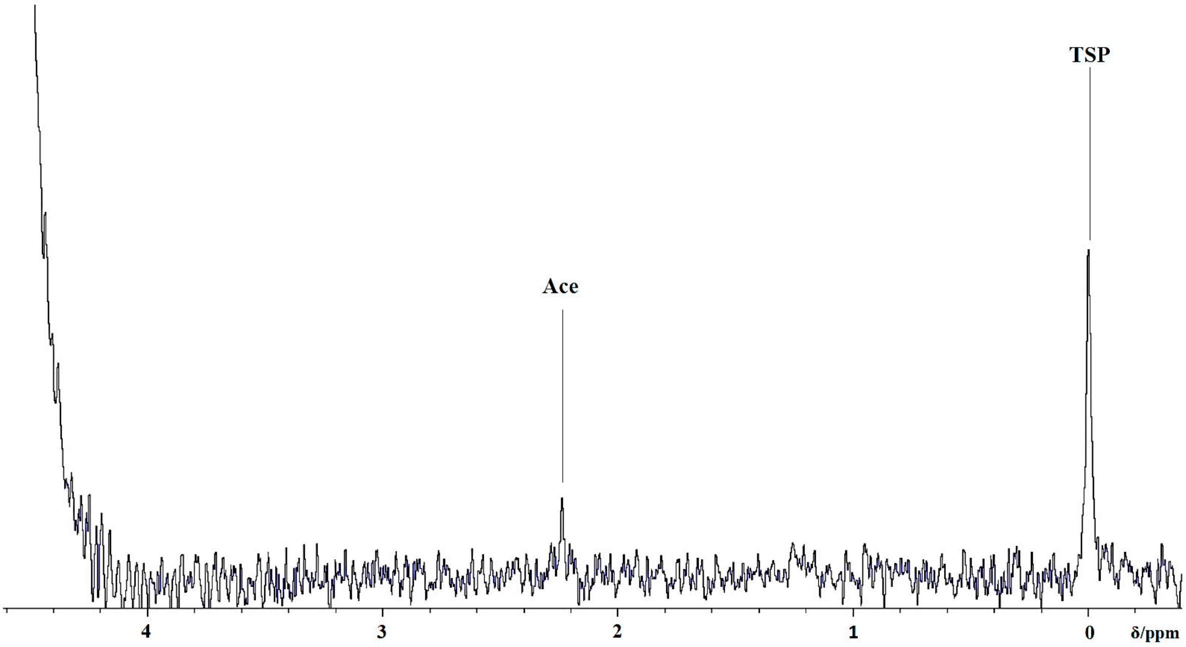

Figure S2: LF 60 MHz benchtop ^{1}H NMR spectrum of a 25 µmol./L acetone calibration standard. Abbreviations: Ace, acetone-CH_3's; TSP, TSP-Si(C<u>H</u>$_3$)$_3$. The STN value for the acetone-CH_3 functions' singlet resonance was found to be 8.8. Spectra were acquired by the methods outlined in section 3.1. For this sample we performed 512 scans (no dummy scans) with acquisition and repetition times of 6.4 and 15 s respectively, and a pulse angle of 90°. A typical spectrum is shown.

SECTION S3: LF 60 MHz ^{1}H NMR calibration standard curves for selected urinary biomolecules

(a)

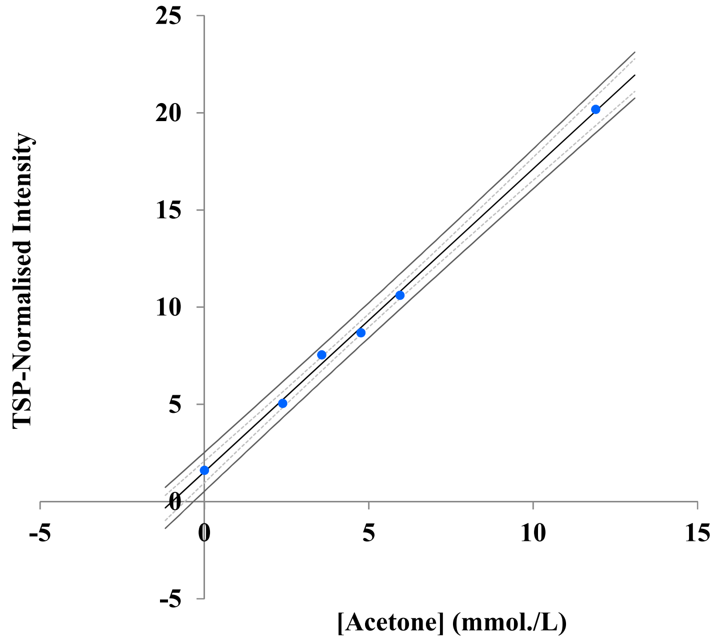

(b)

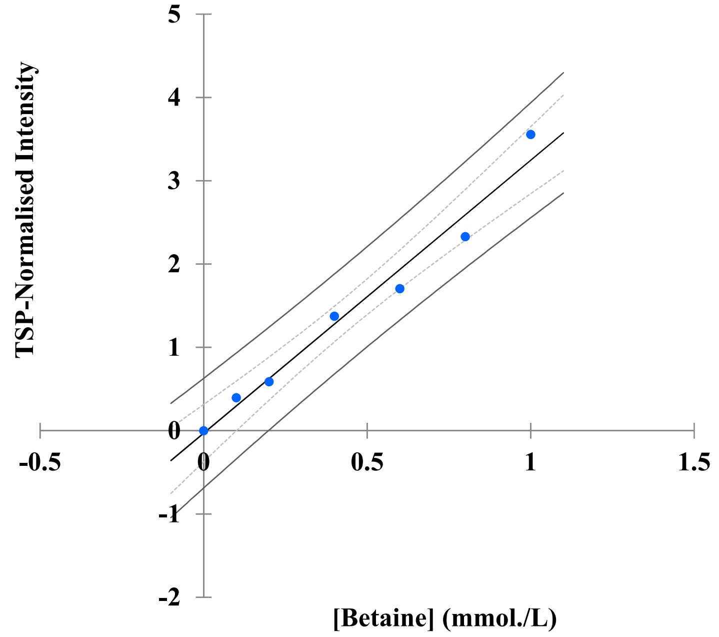

(c)

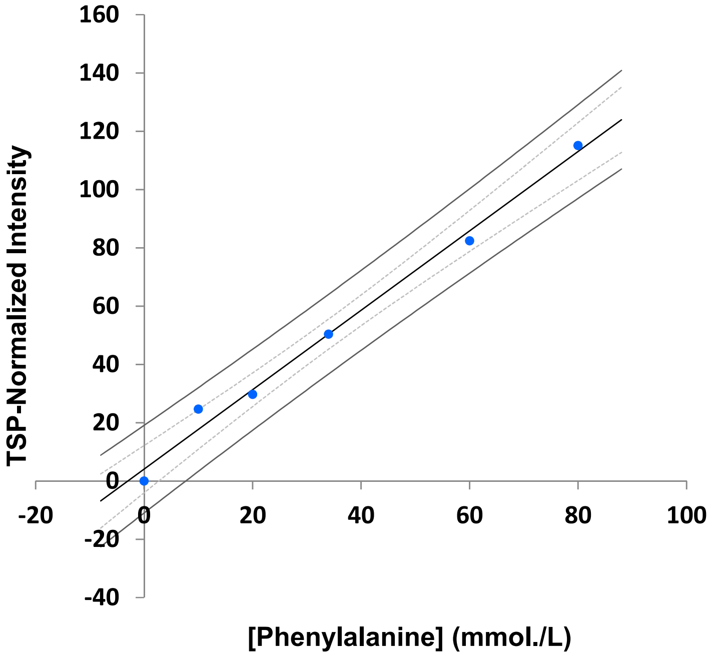

Figure S3. Calibration plots of TSP-normalised resonance intensities *versus* concentrations for **(a)** acetone (*s*, δ = 2.245); **(c)** betaine (*s*, δ = 3.25 ppm); and **(d)** phenylalanine (combined total aromatic ring multiplets, δ = 7.30-7.45 ppm). Correlation coefficient (r) values for plots **(a)**, **(b)**, **(c)** and **(d)** were 0.9991, 0.9870 and 0.9953 respectively. For **(a)**, increasing added concentrations of acetone were 'spiked' into a healthy human urine control sample, whereas the plots shown for betaine and alanine in (b) and (c) respectively were prepared in aqueous solution containing *ca.* 10% (v/v) $^{2}H_2O$ as outlined in the main article file. Abbreviations: - - - - - , 95% confidence intervals for means; ---------, 95% confidence intervals for observations.

Section S4: Spectrophotometric GOD-PAP determinations of urinary glucose concentrations

In this spectrophotometric method, the GOD-PAP reagent allows glucose to be determined via its enzymatic oxidation by glucose oxidase. The hydrogen peroxide (H_2O_2) formed reacts with phenol, a reaction catalysed by peroxidase and 4-aminophenazone to form a red-coloured quinoneimine dye chromophore as indicator. The GOD-PAP reagent and buffer (Randox Laboratories Ltd, Northern Ireland) was reconstituted according to the manufacturer's instructions. Separate standard glucose solutions (Sigma Aldrich, UK) in the range 0.50 mg/mL (2.77 mmol./L) to 2.5 mg/mL (13.8 mmol./L) were volumetrically prepared in 0.90% (w/v) NaCl for a calibration curve. 0.50 mL of GOD-PAP reagent was added to polystyrene cuvettes (1.0 mL path length, Fisher Scientific) and incubated at 37°C (Stuart Scientific Incubator SI 19, UK) for 10 min. Subsequently, 5 μL aliquots of each standard glucose solution was then added to the cuvettes which were then returned to the

incubator at 37°C for a further 10 min. The cuvettes were then shaken to ensure homogeneity and then incubated for a further 10 min. Solutions were analysed spectrophotometrically at a wavelength of 510 nm (Evolution 60S, Thermo Scientific, UK). For the determination of glucose concentrations from urine samples, the same method was employed with 5 μL of urine mixed with a 500 mL volume the of GOD-PAP reagent.

A linear calibration curve for glucose standards 0.50 mg/mL (2.77 mmol./L) to 2.5 mg/mL (13.8 mmol./L) was obtained, with a R^2 value of 0.990 which was employed for determining glucose concentrations in all urine samples available for testing.

Section S5: Plots of urinary glucose concentrations determined by LF 60 MHz ^{1}H NMR analysis *versus* those determined by (a) HF 400 MHz ^{1}H NMR analysis; (b) the spectrophotometric GOD-PAP method; and (c) a simple visual colourimetric dipstick approach.

(a)

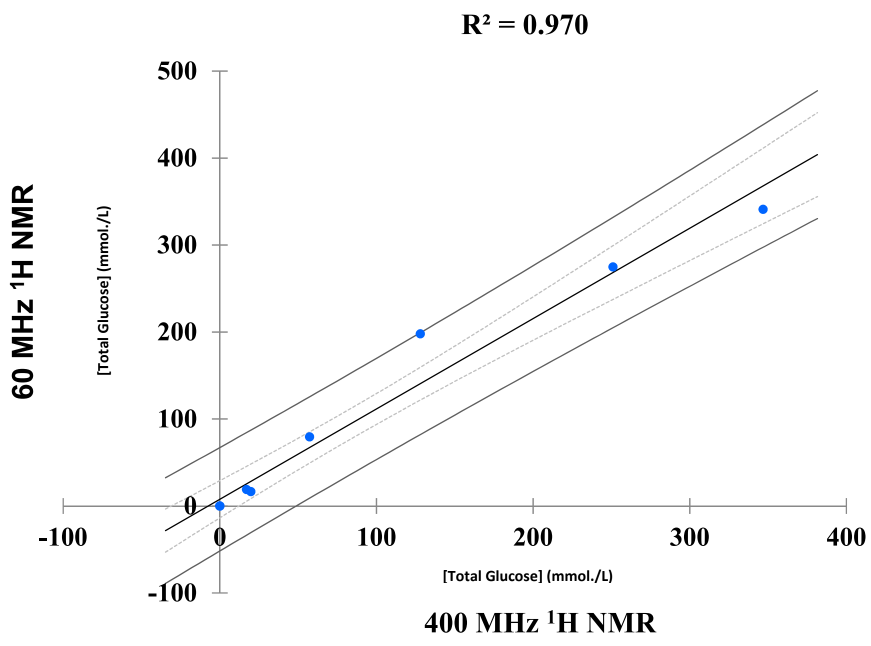

(b)

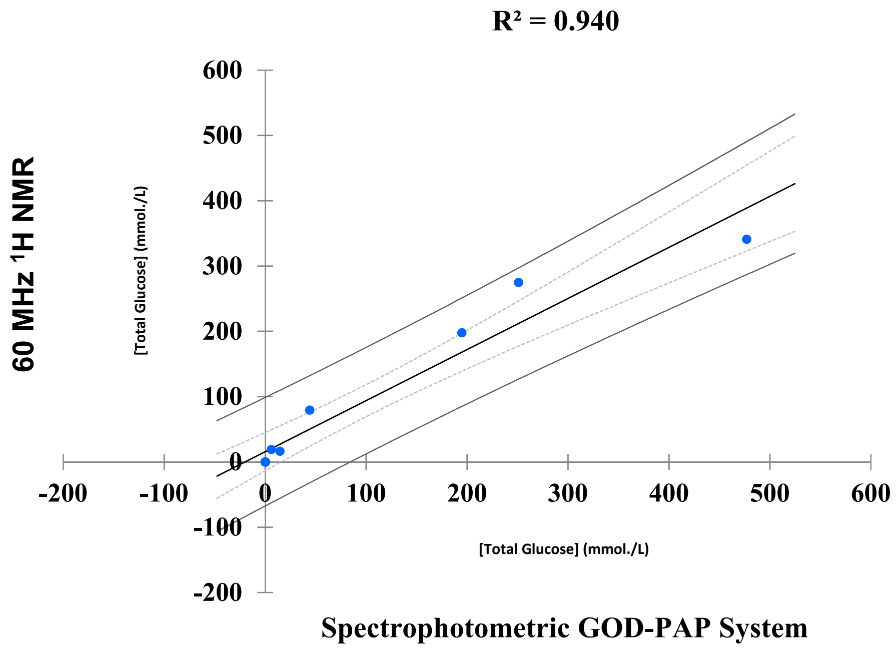

(c)

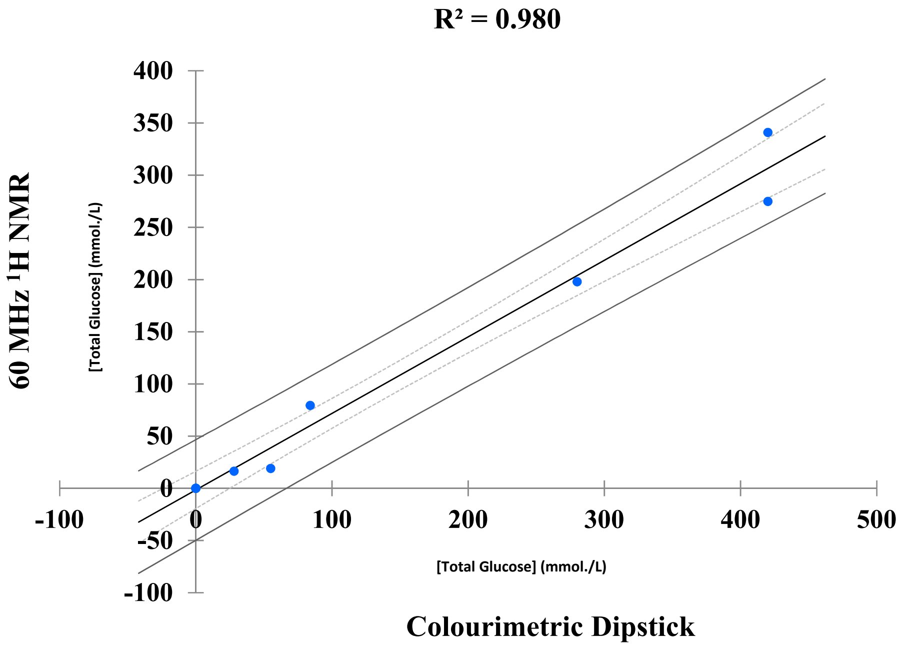

Figure S5. Plots of LF 60 MHz ^{1}H NMR-determined total urinary glucose concentrations versus those obtained from analysis by (a) 400 MHz ^{1}H NMR; (b) Spectrophotometric

glucose oxidase-peroxide/4-aminophenzone/phenol method; (c) Dipstick visual colour testing system.

Section S6. Random Forest-Metabolomics Classification of the ^{1}H NMR Profiles of Type 2 Diabetic and Healthy Control Participants

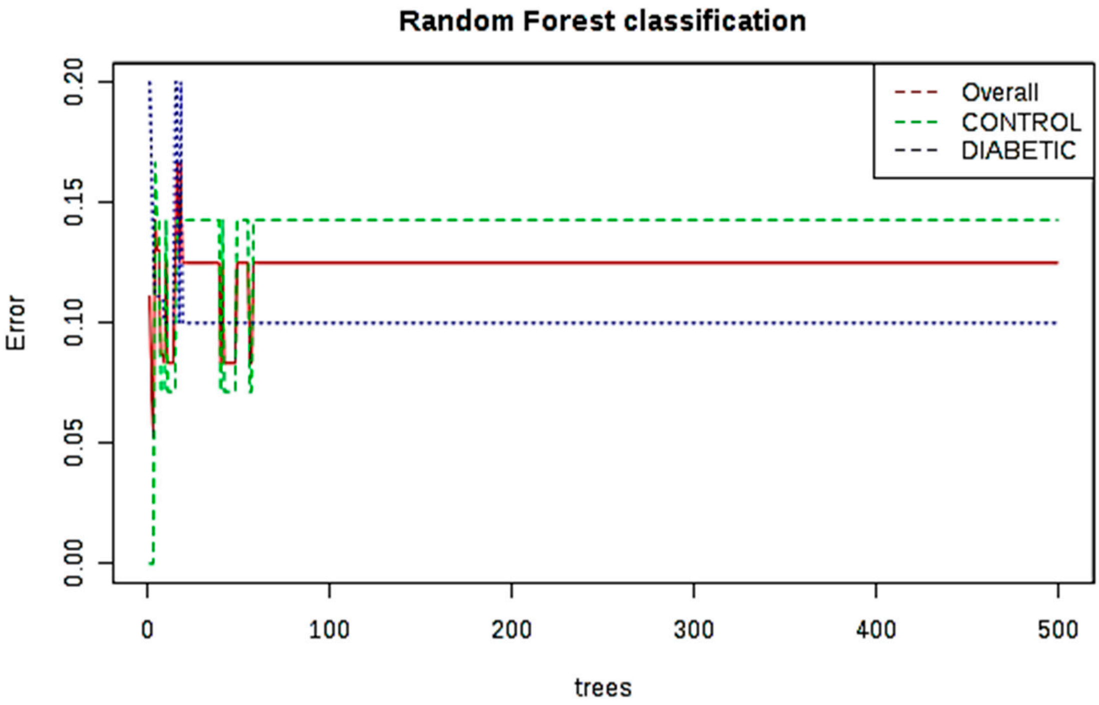

Figure S6. Cumulative error rates expressed as a function of number of trees incorporated within the Random Forest (RF) classification model applied. The red line represents the overall error rate, whereas the blue and green lines show those for the type 2 diabetic and healthy control classifications.

The RF technique was employed for classification and variable selection using the randomForest *R* package, with 500 trees and 7 predictor variables selected at each node subsequent to tuning. Datasets were randomly split into training and test sets containing approximately two thirds and one third of them respectively. The training set was used to build the RFs model and obtain an out-of-the-bag (OOB) error value in order to assess the performance of the classification.

Section S7: LF 60 MHz single-pulse ^{1}H NMR spectral profile of human blood plasma

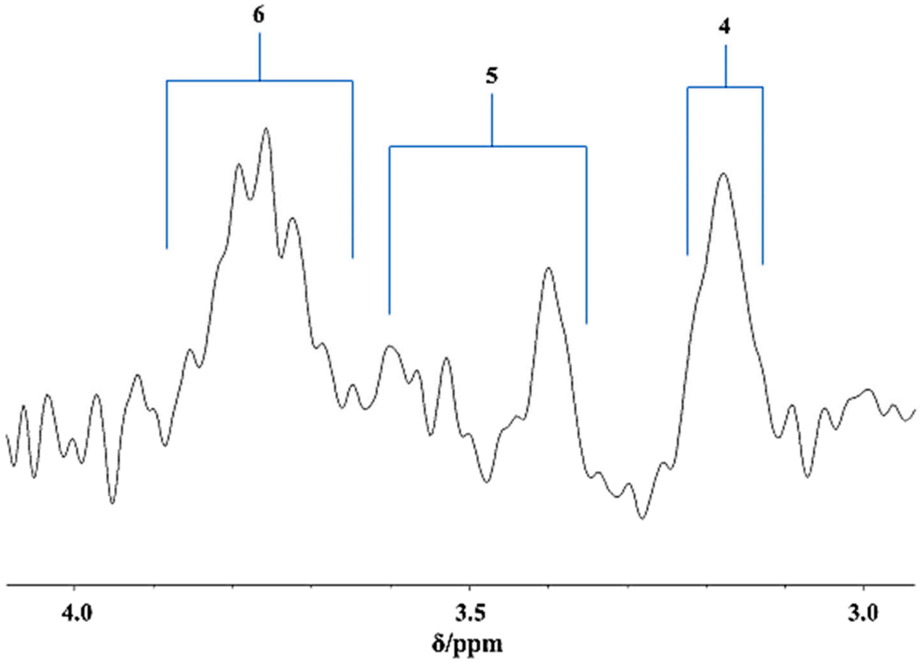

Figure S7. Partial (2.90-4.10 ppm region of) the ^{1}H NMR profile of human blood plasma acquired on a LF 60 MHz benchtop spectrometer. Assignments: 4, 5 and 6 correspond to the C2-H to C6-H_2 bulk carbohydrate ring protons of α- and β-glucose, according to their specification in the Figure 8 legend; a range of further, less intense ^{1}H NMR resonances arising from other biomolecules are also readily observable in this 'carbohydrate region' of HF ^{1}H NMR spectra (i.e. ≥ 400 MHz) acquired on such samples, but only the most intense (glucose) signals are predominantly visible at 60 MHz operating frequency. Samples were prepared for analysis by the treatment of 600 μL of heparinised blood plasma with 65 μL of $^{2}H_2O$ containing 0.05% (w/v) TSP, and 50 μL of a 1.00 mol./L phosphate buffer solution (pH 7.00) containing 0.40% (w/v) sodium azide. ^{1}H NMR spectra were acquired with acquisition and repeat times of 4.6 and 15 s respectively, a pulse angle of 90°, and presaturation of the intense H_2O/HOD resonance. A total of 2,048 scans were acquired. A typical spectrum is shown.
